# Usefulness of medicine screening tools in the frame of pharmaceutical post-marketing surveillance

**DOI:** 10.1371/journal.pone.0289865

**Published:** 2023-08-11

**Authors:** Christelle Ange Waffo Tchounga, Pierre-Yves Sacré, Raffaella Ravinetto, Marya Lieberman, Patient Hamuli Ciza, Rose Ngono Mballa, Eric Ziemons, Philippe Hubert, Roland Djang’eing’a Marini

**Affiliations:** 1 Department of Pharmacy, Laboratory of Pharmaceutical Analytical Chemistry, University of Liege (ULiege), CIRM, Liège, Belgium; 2 Faculty of Medicine and Biomedical Sciences, University of Yaoundé I, Yaoundé, Cameroon; 3 Department of Pharmacy, University of Liege (ULiege), CIRM, Research Support Unit in Chemometrics, Liège, Belgium; 4 Department of Public Health, Institute of Tropical Medicine Antwerp, Antwerp, Belgium; 5 School of Public Health, University of the Western Cape, Cape Town, South Africa; 6 Department of Chemistry and Biochemistry, University of Notre Dame, Notre Dame, IN, United States of America; 7 Faculty of Pharmaceutical Sciences, University of Kinshasa, Lemba, Kinshasa, Democratic Republic of the Congo; 8 Laboratoire National de Contrôle des Médicaments et Expertise (LANACOME), Yaoundé, Cameroon; Kohat University of Science and Technology (KUST), PAKISTAN

## Abstract

The negative consequences of Substandard and falsified (SF) medicines are widely documented nowadays and there is still an urgent need to find them in more efficient ways. Several screening tools have been developed for this purpose recently. In this study, three screening tools were used on 292 samples of ciprofloxacin and metronidazole collected in Cameroon. Each sample was then analyzed by HPLC and disintegration tests. Seven additional samples from the nitro-imidazole (secnidazole, ornidazole, tinidazole) and the fluoroquinolone (levofloxacin, ofloxacin, norfloxacin, moxifloxacin) families were analyzed to mimic falsified medicines. Placebo samples that contained only inert excipients were also tested to mimic falsified samples without active pharmaceutical ingredient (API). The three screening tools implemented were: a simplified visual inspection checklist, a low-cost handheld near infrared (NIR) spectrophotometer and paper analytical devices (PADs). Overall, 61.1% of the samples that failed disintegration and assay tests also failed the visual inspection checklist test. For the handheld NIR, one-class classifier models were built to detect the presence of ciprofloxacin and metronidazole, respectively. The APIs were correctly identified in all the samples with sensitivities and specificities of 100%. However, the importance of a representative and up-to-date spectral database was underlined by comparing models built with different calibration set spanning different variability spaces. The PADs were used only on ciprofloxacin samples and detected the API in all samples in which the presence of ciprofloxacin was confirmed by HPLC. However, these PADs were not specific to ciprofloxacin since they reacted like ciprofloxacin to other fluoroquinolone compounds. The advantages and drawbacks of each screening tool were highlighted. They are promising means in the frame of early detection of SF medicines and they can increase the speed of decision about SF medicines in the context of pharmaceutical post-marketing surveillance.

## Introduction

Substandard and falsified (SF) medicines cause harmful consequences that severely affect health systems and people’s lives. To address this issue, it is imperative to avoid their entry in the supply chain and to remove as quickly as possible the ones that may have entered it. These immediate actions can be taken by the pharmaceutical regulatory authorities, but they rely on the rapid detection of SF medicines [1,2]. Thus, pharmaceutical quality control is a critical tool for essential regulatory functions [3,4]. The World Health Organization (WHO) has developed a Global Benchmarking Tool to assess the strengths and weaknesses for each regulatory function, i.e. national regulatory system, registration and marketing authorization, vigilance, market surveillance and control, licensing establishments, regulatory inspection, laboratory testing, clinical trials oversight and, for overseeing biological products, National Regulatory Authority (NRA) lot release. For “market surveillance and control”, one may divide the quality control of medicines in two main phases: (*i*) a field inspection phase that requires fast and easy to implement testing of the samples, and (*ii*) a confirmatory phase that requires comprehensive laboratory testing of the suspect samples to confirm their status.

The confirmatory phase implies the control of several quality attributes of the medicines as recommended by some specific monographies or validated methods such as the identification of the active pharmaceutical ingredient (API), its assay, a dissolution test, evaluation of the uniformity of dosage units, and the control of impurities (identification and/or quantification) [5]. Besides these usual tests, several specific tests may be required depending on the nature of the medicines, e.g., modified release tablets needs specific dissolution testing, parenteral preparations need sterility and endotoxin testing etc. Confirmatory tests require multiple analytical techniques such as thin layer chromatography (TLC), gas chromatography (GC), high-performance liquid chromatography (HPLC) coupled to several detectors (UV, MS, fluorescence, chemiluminescence), capillary electrophoresis (CE), and UV-visible, Raman, and mid and near infrared (NIR) spectroscopies [6–8].

Most of these confirmatory techniques are expensive and require costly reagents and maintenance. More than 70% of the pharmaceutical regulatory systems are estimated to lack sufficient means to guarantee the quality of their medicines [9]. This situation is most apparent in resource-limited settings, where 56.3% of incidents involving substandard medicines were reported, and where very few laboratories are able to reach international standards [10,11]. The difficulties related to the implementation of laboratory analysis in low-resource countries are multiple: instrument failure, long delivery times for spare parts, power and supplies shortage, high-cost of solvents and weak technical capacities [10,12]. Even if confirmatory techniques are available, they are generally not compatible with the field inspection phase especially in remote and resource limited locations; they require controlled laboratory facilities and highly-trained staff. To circumvent these limitations, many authors have advocated for the implementation of affordable and simple screening techniques, especially those that do not need laboratory facilities or electricity, require little or no maintenance, and can be conducted by operators after minimal training [1,10,13].

Several inspection strategies and techniques have been proposed for field use, including visual inspection, handheld or portable vibrational spectroscopy techniques (NIR and Raman), the GPHF-Minilab using TLC and disintegration test, and paper analytical devices (PADs) [8,10,14,15]. Due to the wide variety of ways that pharmaceutical products can fail quality standards, the combination of several techniques is highly recommended to detect substandard or falsified medicines and to obtain an accurate final answer [8,16,17]. Several authors have combined screening and confirmation methods for the detection of SF medicines [1,18–24] and recently, others attempted to evaluate the practical use of different handheld screening tools on the field [25–28].

In this study, three different affordable screening tools were used to analyze ciprofloxacin hydrochloride and metronidazole tablet samples from different brands collected during a field study [29] and their advantages and drawbacks have been compared. These screening tools were: a simplified checklist for visual inspection test [15], a low-cost NIR handheld reflectance device, and paper analytical devices (PADs). They were chosen because of their low-cost, speed of analysis, ease of access, availability, and applicability to the dosage form of the studied samples (tablets). Ciprofloxacin and metronidazole were chosen because, according to previous studies, they were found among the most available and most used on the African pharmaceutical market. Tablet dosage form was chosen as it was, according to some authors, the most targeted by falsifiers [30,31].

The packaging and tablets for each sample were first analyzed visually using a simplified checklist. Tablets were removed from the blister packs and analyzed with the NIR device, and were then crushed for application to the PADs device. Since specific PAD reaction tests have not been developed for metronidazole yet, PADs were used only on the ciprofloxacin samples.

The objective was to maintain two levels of analysis: (i) visual and (ii) identification of APIs, in order to assess the performances of some low-cost and easy to use screening tools for the quick detection of SF medicines in the fame of post-market surveillance in resource-limited contexts, especially in Cameroon.

## Material and methods

### Samples

The samples collected in Cameroon were divided in three groups:

- 56 independent samples constituted by 28 samples (7 brands, 24 different batch numbers) of ciprofloxacin and 28 samples (8 brands, 25 different batch numbers) of metronidazole collected during a preliminary phase by some mystery shoppers in licensed pharmacies selected conveniently in the cities of Yaoundé and Douala from September 2018 to April 2019. These samples (described in S1 Table) were selected because they represent most brands present on the Cameroonian pharmaceutical market (seven brands of ciprofloxacin and seven brands of metronidazole). They were assayed by HPLC and were found compliant regarding the presence of the correct API in the correct amount (specifications 90.0%-110.0% of declared content). They were used to train the user to the different tools, to select the optimal measurement parameters of the NIR device and to build the models which would be used for NIR analysis. Indeed, for NIR device, it is mandatory to perform a calibration phase where identification models (one-class classifier models) are built before going on the field and these samples were used for this purpose.
- 292 samples (150 ciprofloxacin samples and 142 metronidazole samples) collected during a prevalence study described elsewhere from December 2019 to December 2020 [29].
- Four samples (brands) containing fluoroquinolone substances different from ciprofloxacin, 3 samples (brands) containing nitro-imidazole substances different from metronidazole were collected by some mystery shoppers in licensed pharmacies selected conveniently in the cities of Yaoundé and Douala, and ten placebo tablets prepared with a mixture of talc, starch, silicone dioxide and magnesium stearate to mimic samples without API. These samples are detailed in S2 Table.

### Screening tools

The screening tools (see Table 1) were:

- Simplified checklist for visual inspection test,
- Low-cost NIR handheld device,
- Paper analytical devices (PADs).

These screening tests were entirely performed in Cameroon in the collection cities by both laboratory technicians and high-trained staff near the collection sites, in rooms having access to electricity.

For the PADs and the simplified checklist for visual inspection, the training was performed in 3 steps:

1. Explanation of the test and demonstration of an analysis to the trainee
2. Analysis of a sample by the trainer in parallel with the trainee
3. Analysis of a sample by the trainee and confirmation of the results by the trainer.

This formation can be performed in a couple of hours for both PADs and visual checklist.

All the results obtained were eventually confirmed by highly-trained staff in order to ensure that there is no bias in the sensitivity and specificity results presented in this article.

For the NIR analyses, all analyses were performed by the highly trained staff.

**Table 1:** Description of screening tools deployed.

Screening tool	Purpose	Cost	Description	Handheld?	Decision criteria
**Simplified visual inspection Checklist**	Visual inspection	NA*	26 yes or No questions	Yes	Depending on Risk analysis
**NIR-S-G1**	Identification of API	~1600$	Low cost NIR dispersive spectrometer 900-1700nm	Yes	Chemometric tools (DDSIMCA models)
**PAD**	Identification of API	3$	Paper based color test	Yes	Comparison with a typic card of ciprofloxacin

*NA = Not applicable.

#### Simplified visual inspection checklist

The simplified visual inspection checklist used in this study and also applicable to non-research settings is described by Schiavetti *et al*. [15]. This checklist focuses on the packaging, identity, traceability, and physical appearance of the samples and is intended to detect visual and physical non-conformities on the samples. Originally developed for four different pharmaceutical forms (powders for suspension and syrup; tablets; sterile liquids and powders for injection), it comprises 26 yes/no questions. Since all samples of the present study were in tablet form, only the 16 questions related to tablets were considered. To each yes response, a score ‘A’ was attributed and to each no response, a score ‘B’ or ‘C’ was attributed, depending on the type of the non-conformity. Based on the total score, three final statuses may be assigned to the samples according to the likely level of risk for the patient. The score must be assigned by the researcher (or, outside research, by the healthcare staff) who made the visual assessment, based on detailed, published instructions [15]. In case of doubts, a risk-based assessment is done with the prescriber or, in any case, with a medical doctor, to reach a final decision.

- Status ‘A’: “reasonably safe for dispensing” samples since no visible non-conformity has been identified.
- Status ‘B’: “dispense with explanations” if minor non-conformities have been identified, but they are thought not to compromise the safety or efficacy of the medicine. This is the case if, for instance, some attributes required for the identification, conservation, or traceability of the medicine are missing on the secondary packaging only; or, if the secondary packaging only is damaged; or if an appropriate dosing device is lacking.
- Status ‘C’: “quarantine and make a risk-benefit evaluation before dispensing” if non-conformities are identified that are likely to compromise the safety or efficacy of the medicine. This is the case, for instance, if the primary packaging is damaged; or, if key-information for the identification, shelf life or preparation of the suspension is completely missing; or, if there are visible non-conformities (i.e. clumps, stickiness, heterogeneous colour, residual powder).

The final status of a sample corresponds to the lowest status obtained. If a sample had at least one status ‘B’ for a question, its final status remained B. The same is true for status C even if for other questions it had status ‘A’ or ‘B’. Therefore, samples with status ‘A’ were those without any non-conformities. The samples with status ‘C’ were considered non-compliant according to the visual inspection test as “they can surely compromise the safety or efficacy of the medicine” and those with status ‘B’ were considered suspicious. All the checklist’s results were checked by a pharmacist. Differently from other tools used in this research, the nature of the visual inspection does not allow a calculation of sensitivity or specificity of its findings [15].

#### Near Infrared (NIR) spectroscopy and chemometric analysis

*Instrumentation and data acquisition*. The NIR spectrophotometer used was a low-cost handheld reflective NIR spectrometer (NIR-S-G1, Innospectra Corp., Taiwan). A single device was used either in Belgium and in Cameroon. The NIR-S-G1 was controlled by a laptop with the ISC-NIRScan-GUI software (version 3.5.7) in order to collect spectra. Each spectrum corresponds to an average of 6 scans in the range of 900-1700nm. The lamp was turned ON before starting the analysis (pre-heating phase) until a stable detector temperature was reached (~60°C in Cameroon and ~ 55°C in Belgium and humidity ~10% RH in Cameroon and ~0% RH in Belgium) were reached inside the device. This preheating phase is important since the single pixel InGaAs detector of the NIR-S-G1 device has a high sensitivity to temperature at the edges of the spectral range (below 950 and above 1600 nm). Therefore, the pre-heating enabled the analysis of samples with a negligible spectral drift due to the temperature change inside the device. A new background was acquired with the same parameters before each sample analysis with a Spectralon® white diffuse reflectance standard (99%). More specifications about spectra acquisition are available in S1 File.

All spectra were collected in reflection mode on the tablets before any analysis requiring tablet destruction (HPLC, disintegration test, or PAD). All the tablets were scanned outside the blisters to avoid the influence of the primary packaging and to be able to analyze tablets in aluminum-aluminum blisters. The spectra of the calibration phase were acquired in Liege (Belgium) during the preparation phase of the field study. Ten spectra were taken per sample i.e. one spectrum per tablet on ten different tablets per sample. When a tablet showed a flat side, this one was privileged for taking spectra. As these NIR spectra cannot be interpreted directly, chemometric tools (performed by experts) are needed in order to detect the presence or not of the correct APIs in drug samples.

#### Chemometric tools

*Preprocessing*. The preprocessing applied on all spectra were the first Savitzy-Golay derivative (2^nd^ degree polynomial and window size of 15) and Standard Normal Variate (SNV). The selected spectral range was 961-1624nm.

*One-Class Classifier (OCC) model*. Data-Driven Soft Independent Modeling of Class Analogy (DD-SIMCA) is a principal component analysis-based One-Class Classifier (OCC) algorithm [32]. It was used to identify the presence of ciprofloxacin and metronidazole in samples and to discriminate these drugs from the other active pharmaceutical ingredients (APIs) belonging to fluoroquinolone or nitro-imidazole families.

*A priori* models were calibrated using the spectra measured in Belgium on ciprofloxacin and metronidazole samples collected during a preliminary study (see section 1). The spectra obtained were split into calibration and validation sets using the Kennard-Stone algorithm [33,34] keeping replicates together: the spectra of 19 samples (68%) were placed in the calibration set and those of 9 other samples (32%) in the validation set.

Afterwards, *a posteriori* models were built with newly selected datasets from the whole recoreded spectra (preliminary study + field study). The training dataset is constituted of 28 samples selected with the Kennard-Stone algorithm (keeping replicates together) to uniformly span the spaces of samples for ciprofloxacin and metronidazole. The test set is constituted from the remaining samples (150 for ciprofloxacin and 142 for metronidazole). The training dataset is eventually split into calibration (19 samples) and validation set (9 samples) by Kennard-Stone algorithm and is used to tune the model parameters. Spectra of four samples with different APIs belonging to the fluoroquinolone family (norfloxacin, ofloxacin, levofloxacin, moxifloxacin) were used to check the specificity of the OCC ciprofloxacin model. Spectra of three samples belonging to the nitro-imidazole family (tinidazole, secnidazole, ornidazole) were used to check the specificity of the OCC metronidazole model (S2 Table). The spectra of ten placebo tablets were used to mimic samples without API.

The spectra obtained were preprocessed and projected onto the OCC models.

Samples passing the test (positive identification) were considered as positive and the ones failing the test (negative identification) were considered as negatives.

The performances of the models were evaluated by their sensitivity and specificity. using the following formulas [35]:

$$Sensitivity\;(\%)=\frac{TP}{TP+FN}\times 100$$ (1)

$$Specificity\;(\%)=\frac{TN}{TN+FP}\times 100$$ (2)

Where TP = number of true positives, FN = number of false negatives, TN = number of true negatives, FP = number of false positives.

Confidence intervals at 95% were computed with a bootstrapping strategy described in supplementary data (S1 File). The *a priori* and *a posteriori* models obtained were used as initial ones, enabling model parameters to be set before applying bootstrapping analysis.

As ten tablets were analysed per sample and one spectrum was recorded per tablet, the positive identification of a sample was based on the number of accepted spectra per tablets. In the present manuscript, it was decided to allow a maximum of 4 rejected spectra per sample (6 accepted spectra) for a positive sample identification. This rule may be changed depending on level of risk the analyst may find acceptable.

*Paper Analytical Devices (PADs)*. PADs are paper microfluidic devices which contain 12 lanes, separated by hydrophobic wax barriers, that are impregnated with specific reagents. They are dedicated to detect the presence of the stated API in a sample drug. The sample is applied near the middle of the lanes by smearing powder from a pharmaceutical tablet or capsule across all 12 lanes. The bottom of the card is then dipped into water, which moves up the lanes by capillary action, dissolving and transporting the stored reagents to the powder. These reagents undergo specific color reactions with specific functional groups of APIs or excipients. The outcomes are not simple yes-no tests; some reagents give different colors for different functional groups, or produce colors in different parts of the lane for different test substances [20,24,36,37]. The results of the twelve color tests form a “color bar code” of the sample which can be compared to a reference [24,36]. For the 12-lane PADs used to analyze ciprofloxacin samples, a positive test generates a blue color at the “swipe line” in lane D and an orange color at the top of lane L. Lane D contains an acid that can protonate tertiary amines and the potassium salt of cobalt thiocyanate, which forms a blue, insoluble ion-pair with two equivalents of protonated tertiary amine. Lane L contains iron chloride, which reacts with 1,3 dicarbonyls to form soluble orange complexes similar to iron(III) tris(acetylacetonate). Ciprofloxacin contains both a tertiary amine and a 1,3 dicarbonyl, so it triggers both of these lanes [37].

After analysis, the cards were photographed using a smartphone camera within 5 minutes and the resultant color code was compared to a reference and confirmed by experts from University of Notre Dame. These ciprofloxacin PAD images were stored on Dropbox. As an alternative to visual evaluation of color test results for use by people who are untrained in reading the PADs, and to avoid false positive or negative results due to the subjective assessment of colors, an android phone app (PADreader) was developed [38]. As this was not available at the time the study was carried out, the PADs images stored were photographed from computer monitor before being analyzed by the App. The samples whose cards showed colors different from blue and orange on lanes D and L respectively, were considered suspicious. A suitability test was performed during training and at the start of the experiments with a blank card (with no substance added on the card), one card with ciprofloxacin reference substance and another with ciprofloxacin sample. The ciprofloxacin color code was obtained on ciprofloxacin reference substance cards and samples, and as expected, not on the blank card (S1A–S1C Fig). Samples containing other fluoroquinolones different from ciprofloxacin were also tested before going to the field. Sensitivity and specificity were calculated for PADs for identification purpose in comparison with HPLC assay results performed in the study [29].

### Software

Chemometric analyses were performed using PLS toolbox v.8.7 (Eigenvector Research, Inc.,Wenatchee, WA, USA) and in-house scripts in a MATLAB environment (R2018a) (The Mathworks, Inc., Natick, MA, USA). All information concerning the simplified visual inspection checklist was encoded in Excel 2016 file (Microsoft Corp., Redmond WA, USA). The PADs images were photographed from a computer monitor and analyzed with PADreader 2.4 (Google Play Store, installed Nov 1^st^ 2022) on a Pixel 6a phone running Android version 13. For this study, the drug classifier “fhi360_large_454x454_image” (available in the App) was used; this is a neural net classifier which is trained to differentiate amoxicillin, albendazole, ampicillin, azithromycin, benzyl penicillin, ceftriaxone, chloroquine, ciprofloxacin, doxycycline, isoniazid, pyrazinamide, rifampicin, tetracycline, ethambutol, Rifampicin-Isoniazid-Pyrazinamide-Ethambutol fixed-dose (RIPE), hydroxychloroquine, epinephrine, ferrous sulfate, promethazine hydrochloride, and sulfamethoxazole.

## Results and discussion

### Samples description

The samples involved in this study were collected during a previous prevalence study [29]. The laboratory analysis of the 292 samples (46 different brands of ciprofloxacin and 18 different brands of metronidazole) revealed that 18 were non-compliant. These non-compliant samples were 7 metronidazole, all failing the disintegration test and 11 ciprofloxacin: 5 failed the HPLC assay (dosage < 90%), 5 failed the disintegration test, and 1 sample failed both disintegration and assay tests. In addition to these substandard samples (also called out-of-specifications), 2 were precautionarily considered as possibly falsified according to visual inspection because they lacked key information about the manufacturing company either on the primary or secondary packaging. This information is essential for checking the regulatory status of the product, whether it comes from a licensed or an illegal source, and for doing batch recalls in case of need.

### Simplified checklist for visual inspection test

Based on the simplified visual inspection checklist, as shown on Table 2, 19% of the samples (n = 56) were reasonably safe for dispensing (status ‘A’). For these samples, no visible non-conformities were identified. 85.7% were ciprofloxacin samples (n = 48) and 14.3% (n = 8) were metronidazole samples. Most of the samples (77.0%) had the final status ‘B’ (Metronidazole samples n = 134 and Ciprofloxacin samples n = 91).

**Table 2:** Results of simplified visual inspection checklist.

Questions	Results			Number of samples
	A	B	C	
A.Packaging	Yes	No		
1.Is there an external packaging?	169	123	NA	292
2.Is the external packaging intact?	164	128	NA	292
3.Is the internal packaging intact?	288	NA	4	292
4.Does the internal packaging provide clear information on the storage conditions of the medicine?	84	208	NA	292
**B. Identification** B1. Does the external packaging carry the following information on the outer side?	**A**	**B**	**C**	
	**Yes**	**No**		
5. Name of the active ingredient (s)	169	0	NA	169
6. The amount of active ingredient per dosage unit?	169	0	NA	169
7. The expiry date in an uncoded form (i.e. Exp.Date 06/20, JUN 20)	163	6	NA	169
B2. Does the internal packaging carry the following information on the outer side?	**Yes**	**No**		
8. Name of the active ingredient (s)	292	NA	0	292
9. The amount of active ingredient per dosage unit?	292	NA	0	292
10.The expiry date in an uncoded form (i.e. Exp.Date 06/20, JUN 20)	289	NA	3	292
**C. Traceability** C1. Does the external packaging carry the following information on the outer side?	**A**	**B**	**C**	
	**Yes**	**No**		
11. The name and address of the manufacturer or the company/Person responsible for placing the product on the market?	163	6	NA	169
12. The batch number?	163	6	NA	169
C2. Does the internal packaging carry the following information on the outer side?	**Yes**	**No**		
13. The name and address of the manufacturer or the company/Person responsible for placing the product on the market?	228	64	NA	292
14. The batch number?	286	NA	6	292
**D. Physical appearance**	**A**	**B**	**C**	
15. Have the tablets the same shape, dimensions, color, marks?	291	NA	1	292
16. Are the tablets free from craks, erosion, stains, foreign particles?	290	NA	2	292
**Checklist Final Results**	**A**	**B**	**C**	
	77.0%	19.2%	3.8%	

The main reasons for the ‘B’ status were: lack of secondary packaging, integrity problems on primary and secondary packaging, lack of information about storage conditions on the secondary packaging, lack of expiration date, name, address of the manufacturer, batch number on the secondary packaging (Table 2). According to Schiavetti *et al*., these non-conformities are not susceptible to directly affect the safety and efficacy of the medicines [15]. However, both prescribers and patients should be aware of important information like storage conditions, traceability information and expiry dates. Unfortunately, many patients in resource-limited settings do not receive these information since they buy their medicines without outer packaging, particularly in the informal sector where pharmacists are not present [39]. Traceability information are critical, especially in the case of quality problems or adverse reactions reports within the framework of post-marketing surveillance or pharmacovigilance [24].

The status ‘C’ was obtained for 3.8% of the samples (n = 11) because of physical appearance problems (presence of stains and cracks on the tablets, presence of a half tablet in an intact blister), non-integrity of the primary packaging, lack of expiration date and batch number on the primary packaging (Table 2). They were all ciprofloxacin samples (S2A–S2D Fig). These samples should be quarantined and a risk-benefit evaluation should be made for deciding if they should be dispensed with recommendations or discarded, as according to Schiavetti *et al*., the non-conformities observed can compromise the efficacy and safety of medicines [15]. Furthermore, a complaint should be issued to the concerned suppliers, and the NRA should be informed about the observed non-conformities.

These different findings highlight some problems related to good manufacturing practices (GMP) which can certainly affect the quality of the medicines [15,40]. The status ‘B’ obtained for most of the samples (77.0%) brought to light the structural deficiencies in the Cameroonian pharmaceutical supply chain.

Another finding concerned two samples that were considered falsified in the prevalence study [29] as information about manufacturing company were lacking either on the primary or secondary packaging (S2B Fig). According to the simplified visual inspection checklist, these samples got status ‘B’. However, here we considered that the level of risk should be higher, because of the complete lack of information about manufacturing company [15]. This lack of traceability may be considered as an attempt to obscure the identity and origin/source of the medicine. It is a critical non-conformity that will make it impossible to trace the manufacturer, and it can represent a significant risk for the patient. Mohamed Ali *et al*. reported a similar situation in Sudan confirming the significance of these information for acceptance of pharmaceutical products in a country. They suggested that the status ‘C’ instead of ‘B’ should be assigned to this kind of medicines, making the simplified checklist more efficient in the fight against SF medicines [41].

Overall, among the 18 samples that failed disintegration and/or assay tests, 61.1% (n = 11) also failed the visual inspection test: three with status ‘C’ and eight with status ‘B’. However, 7 of these 18 substandard samples according to disintegration (five samples) and assay tests (two samples), were reasonably safe for dispensing (status “A”) regarding the visual inspection checklist test. As a result, visual inspection and physicochemical non-conformities are not always correlated. Nevertheless, visual inspection allows rapid assessment of some essential medicine quality attributes in the frame of quality control, and is a useful tool in the quality monitoring of medicines either on the field or at central level in routine practice, provided that its limitations are well understood by the users [15,41]. Moreover, the simplified checklist is a very simple and practical tool during the initial phase of a field inspection. While Tack *et al*. have used it to screen the quality of some antibiotic-containing paediatric medicines [42], this is to the best of our knowledge the first time that this simplified visual inspection checklist was systematically applied on solid pharmaceutical dosage forms.

### Near infrared (NIR) handheld device

NIR spectroscopy was used on tablets to confirm the presence of the correct APIs. For this purpose, two OCC models were built: one for ciprofloxacin and another one for metronidazole (target classes). The parameters of these models, their sensitivities and specificities are reported in Table 3.

**Table 3:** Parameters of DD-SIMCA models and performances in terms of sensitivity and specificity.

Metrics	*a priori* Ciprofloxacin model	*a posteriori* Ciprofloxacin model	*a priori* Metronidazole model	*a posteriori* Metronidazole model
**Spectral range**	961–1624 nm	961–1624 nm	961–1624 nm	961–1624 nm
**Preprocessing**	SNV, SG (1, 2,15)	SNV, SG (1, 2,15)	SNV, SG (1, 2,15)	SNV, SG (1, 2,15)
**α**	0.01	0.01	0.01	0.01
**Number of PC**	2	3	2	5
**Median Sn Cal (% of spectra) (95% CI)**	99.5 (97.9–100)	98.4 (96.3–99.5)	97.4 (95.3–99.0)	97.4 (95.3–99.5)
**Median Sn Val (% of spectra) (95% CI)**	96.7 (91.1–100)	94.4 (88.9–97.8)	88.9 (81.1–97.8)	88.9 (80.0–96.7)
**Median Sn test (% of samples) (95% CI)**	98.0 (90.0–100)	100 (99.3–100)	75.4 (68.3–93.7)	100 (97.9–100)
**rejected non-members samples/total number of non-members samples (95% CI)**	5/5 (5–5)	5/5 (5–5)	4/4 (4–4)	4/4 (4–4)

SNV = Standard Normal Variate

SG = Savitzky-Golay derivative (derivative order, polynomial order, window size)

Sn = Sensitivity

CI = confidence interval delimited by the 2.5^th^ and 97.5^th^ percentile of the empirical distribution obtained by bootstrap.

**Fig 1:**
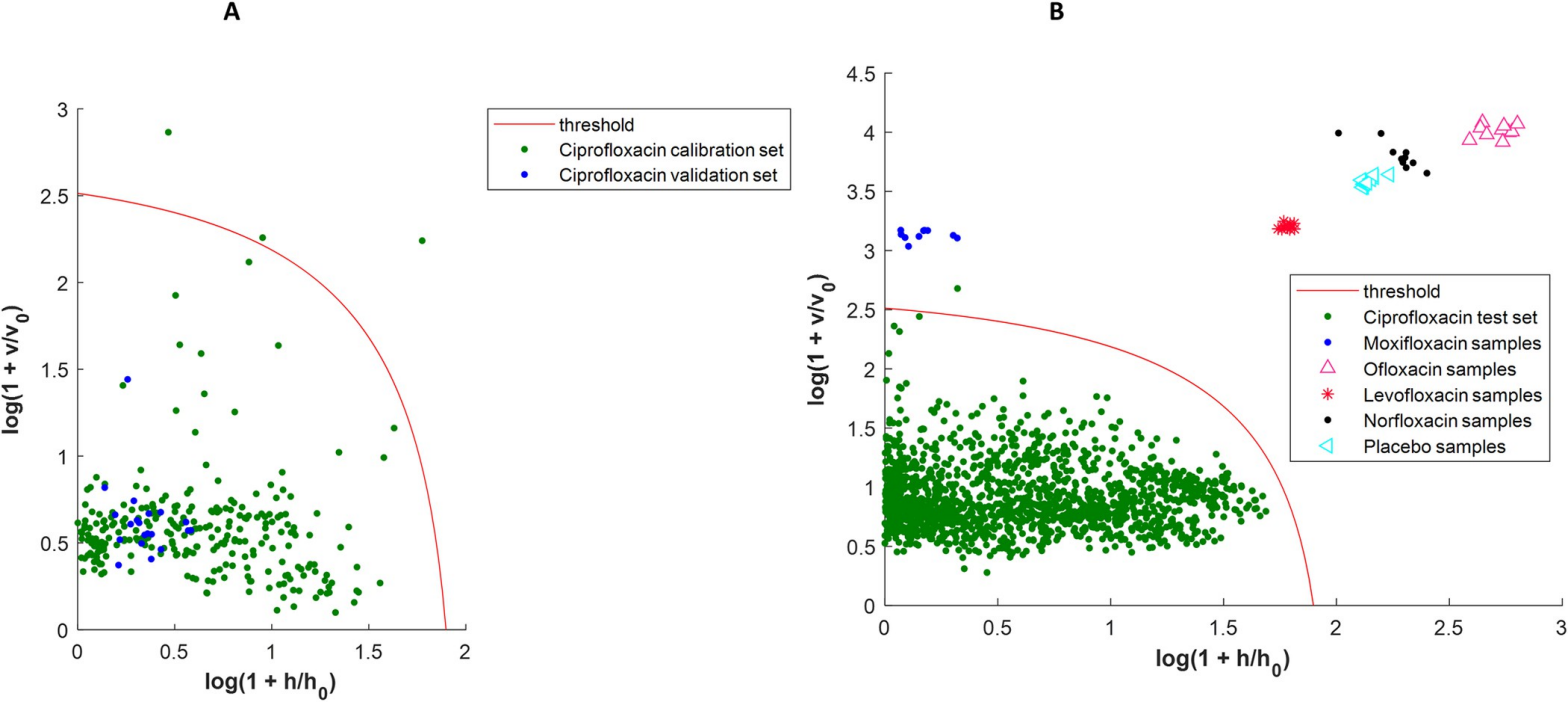
Ciprofloxacin DD-SIMCA acceptance plots (*a priori* model). A: DD-SIMCA model with calibration and validation sets; B: DD-SIMCA model with test sets. For A and B, samples are considered as belonging to the target class if they are below the threshold in the left corner of the plot. This model is the initial *a priori* one before applying bootstrapping analysis.

For the ciprofloxacin *a priori* model, the sensitivities obtained from the calibration datasets was close to the selected α level (99%) with relatively small confidence intervals (CI) [32] (Fig 1A). When projecting the validation samples onto this model, a median sensitivity of 96.7% is observed and the 95% CI ranges from 91.1 to 100%. When the spectra of the 150 ciprofloxacin test samples (1500 spectra) were projected onto the model, the median sensitivity increases to 98.0% but the 95% CI widens (Fig 1B and Table 3).

**Fig 2:**
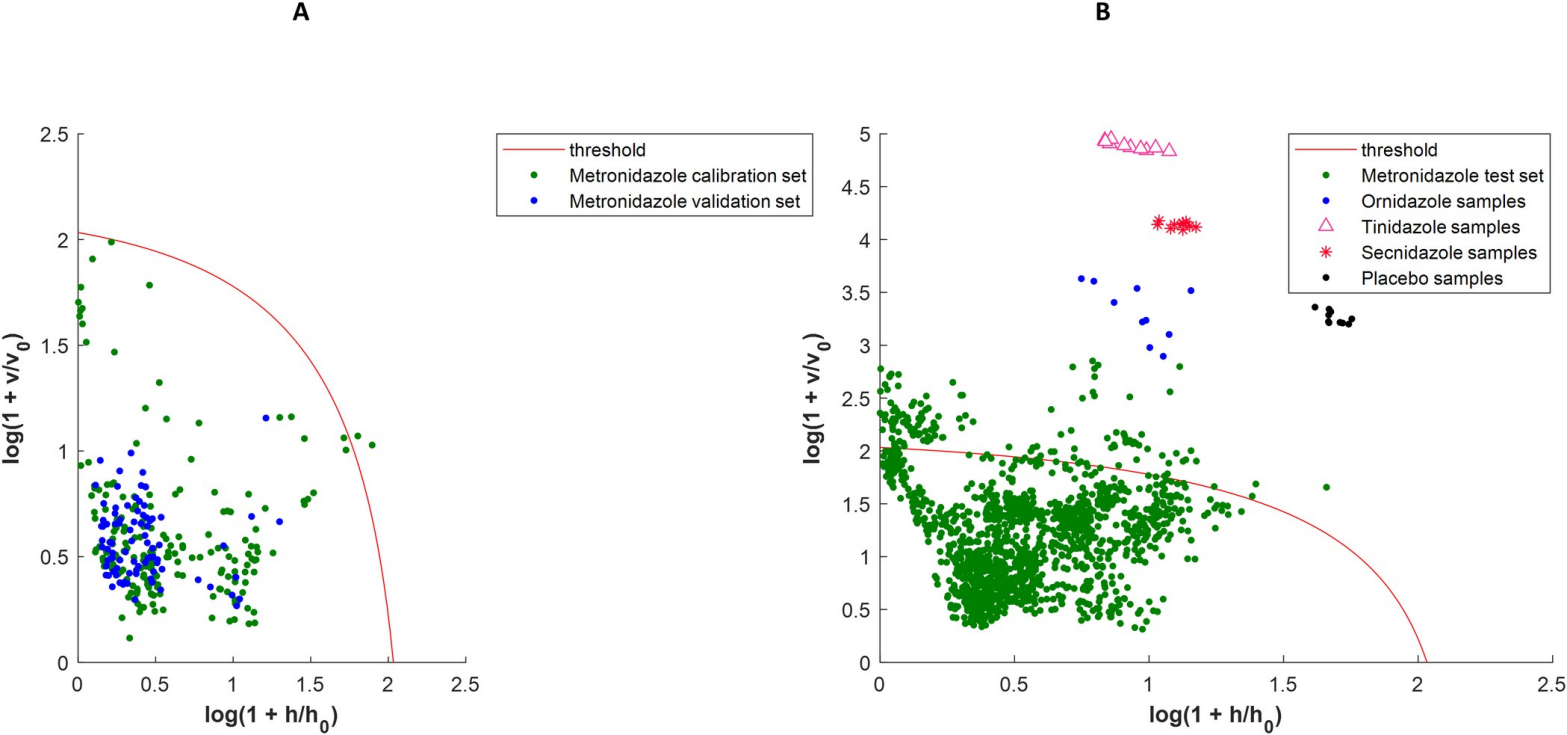
Metronidazole DD-SIMCA acceptance plots (*a priori* model). A: DD-SIMCA model with calibration and validation sets; B: DD-SIMCA model with test sets. For A and B, samples are considered as belonging to the target class if they are below the threshold in the left corner of the plot. This model is the initial *a priori* one before applying bootstrap analysis.

Regarding the metronidazole *a priori* model (Fig 2A), The median sensitivity obtained from the calibration set was 98.4% with a small 95% CI (96.3–99.5%), which is close to the *a priori* sensitivity according to the α selected (99%) [32]. When projecting the spectra from the validation set (9 samples) onto this model, the median sensitivity drops a little and the 95% CI widens in a similar way to the ciprofloxacin model. However, when the spectra collected (18 brands) in Cameroon were projected onto the model (1420 spectra), the median sensitivity drops to 75.4% and the 95% CI ranges from 68.3 to 93.7% which demonstrates a poor performance of the model on the new samples (Fig 2B and Table 3). This low sensitivity brings out the lack of representativity of calibration samples as most of the metronidazole test spectra considered as outliers belonged to 5 different brands not represented in the calibration set (when considering the initial model before bootstrap analysis). Only a few spectra from 7 samples belonging to 2 brands represented in the calibration set were considered as outliers (see S3 Table).

For these *a priori* ciprofloxacin and metronidazole models, one can observe that the extreme plots showed objects outside the tolerance area (S3A and S3B Fig). So, another approach was considered for the two models as the first ones were not quite robust.

A possible explanation for the discrepancy between the preliminary study sample spectra (acquired in Belgium) and the field study spectra (collected in Cameroon), namely the environmental (temperature and humidity) differences between the two sites, was investigated. Regarding the PCA score plots in S4A and S5A Figs, one can see that there is no clear difference between spectra acquired in Belgium or in Cameroon. Therefore, differences in environmental conditions were rejected as explanation of the discrepancies observed between the preliminary and the field study spectra. However, based on the same PCA projections, one may see that the training set based on the preliminary study samples does not span (notably for metronidazole) the space of the collected samples during the field study. This highlights the lack of representativity of the training set. To make a fair estimation of the performance of the NIR device, *a posteriori* models were built from a subset of the whole set of samples (preliminary study + field study) of the same size. This was possible since the presence of API was confirmed in all the test samples by HPLC. The acceptance limits were built with a threshold on the total distance (*α* = 0.01) for both ciprofloxacin and metronidazole OCC models.

**Fig 3:**
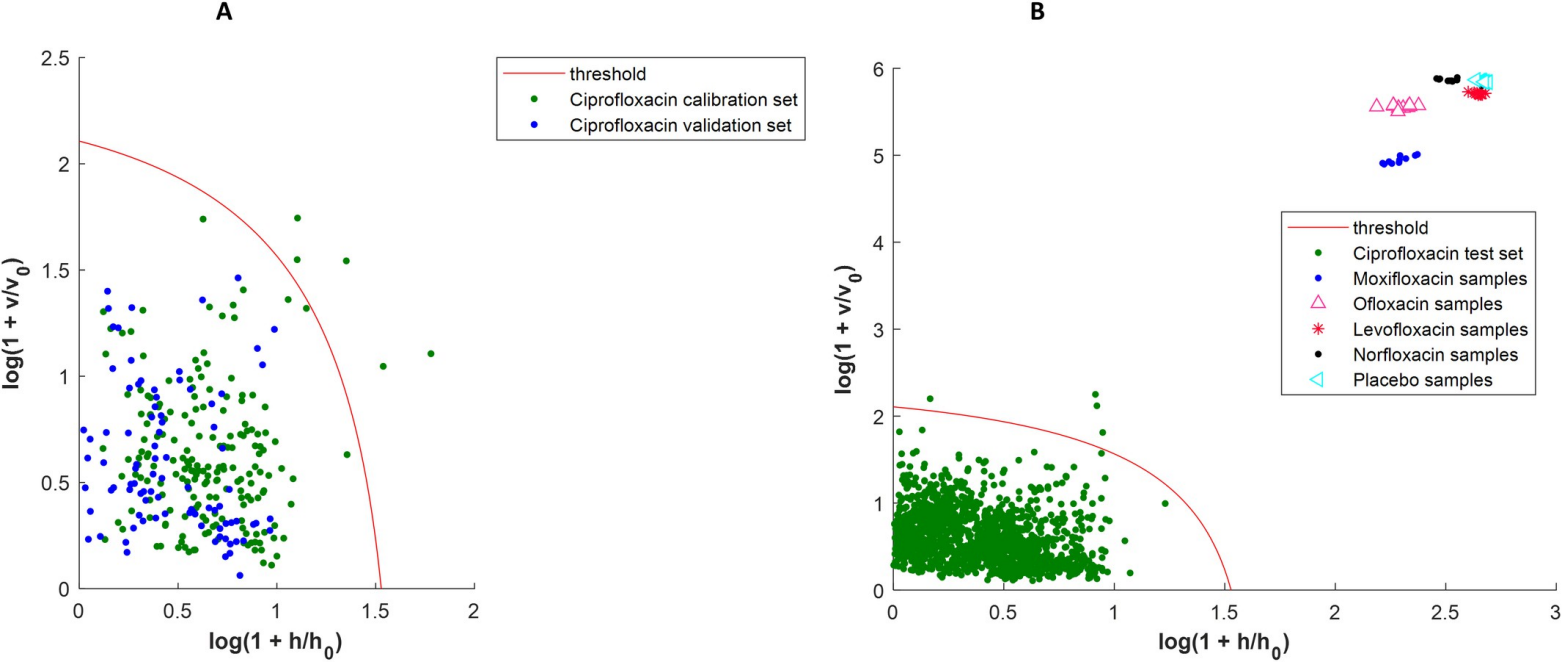
Ciprofloxacin DD-SIMCA new acceptance plots with optimization of the calibration set (*a posteriori* model). A: DD-SIMCA model with calibration and validation sets; B: DD-SIMCA model with test set. For A and B, samples are considered as belonging to the target class if they are below the threshold in the left corner of the plot. This model is the initial *a posteriori* one before applying bootstrap.

The *a posteriori* ciprofloxacin model was built with 3 principal components (Table 3). Five extreme spectra were observed (Fig 3A). The new extreme plot (S3C Fig) showed that there was no more extreme objects in the calibration sets. The results of the bootstrapping analysis showed that for the validation set, the results are nearly the same as for the *a priori* model. However, for the test set, even if the median sensitivity is increased a little, the 95% CI is clearly narrowed. Indeed, for the *a priori* model, the 95% CI was 10.0% wide while for the a posteriori model it is now 0.7% wide which demonstrates an improvement in performances. The fluoroquinolone spectra (moxifloxacin, levofloxacin, norfloxacin, ofloxacin), and placebo samples were also projected onto the ciprofloxacin models to simulate possible substitution with these APIs and test the specificity of the model. All these spectra were correctly discriminated by the two models i.e. a specificity of 100% (Figs 1B and 3B).

**Fig 4:**
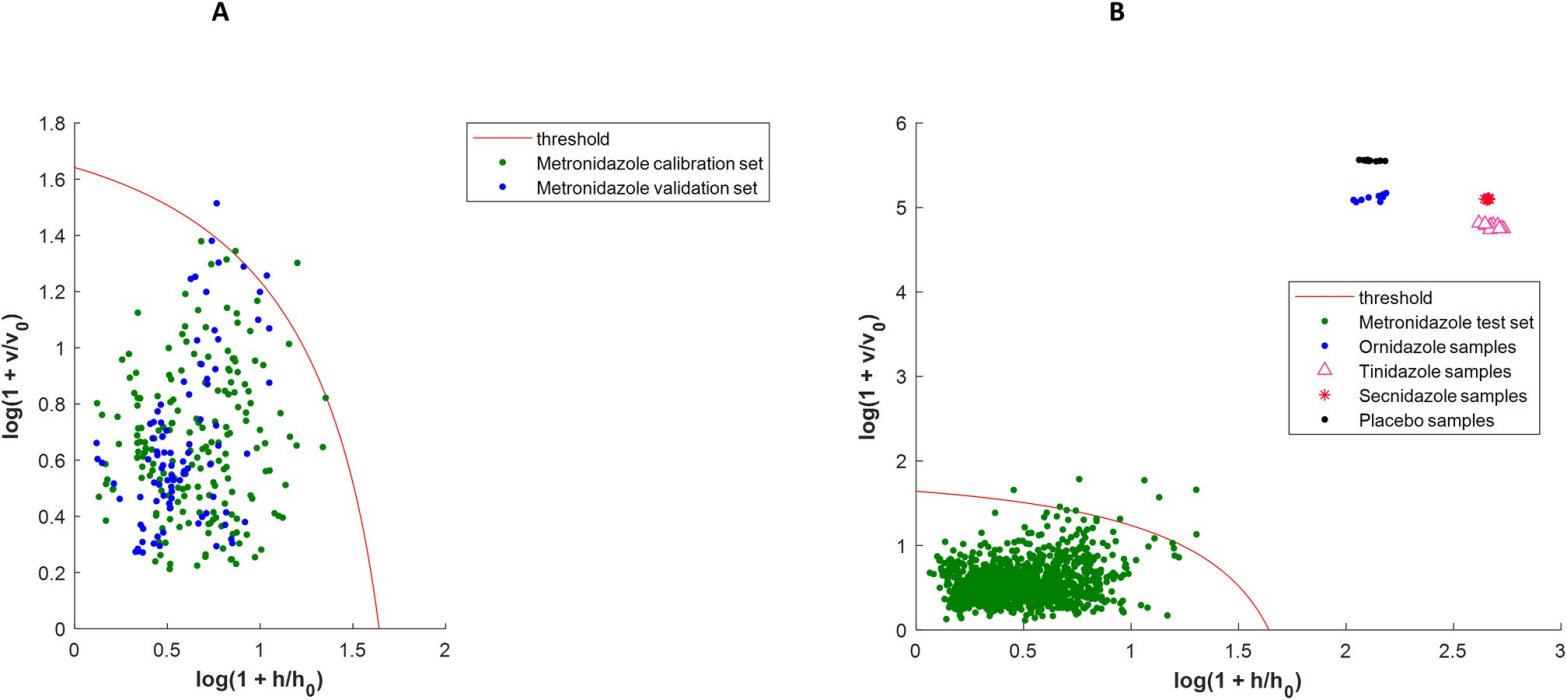
Metronidazole DD-SIMCA new acceptance plots with optimization of calibration set (*a posteriori* model). A: DD-SIMCA model with calibration and validation sets; B: DD-SIMCA model with test sets. For A and B, samples are considered as belonging to the target class if they are below the threshold in the left corner of the plot. This model is the initial *a posteriori* one before applying bootstrap.

The *a posteriori* metronidazole model was built with 5 principal components (Table 3). The extreme plot showed that the calibration set was not contaminated by outliers (S3D Fig). As for the ciprofloxacin, the *a posteriori* model exhibit clearly better performances since its median sensitivity is of 100% (compared to 75.4% for the *a priori* model) and its 95% CI that was 25.4% wide is now only 2.1% wide. As with the ciprofloxacin models, spectra of samples belonging to the nitro-imidazole family (tinidazole, secnidazole, ornidazole), and placebo were projected onto the metronidazole models to simulate possible falsifications (wrong API or lack of API). These spectra were correctly discriminated by the two models (Figs 2B and 4B), as no false positive was detected i.e. a specificity of 100.0%.

Considering specificity results, all OCC models allowed a good discrimination of ciprofloxacin and metronidazole against placebo and other substances having structures close to the target classes, fluoroquinolone and nitro-imidazole samples respectively. These results are similar to those of Ciza *et al*. who found that the application of OCC models to NIR spectra allowed a good discrimination between samples containing different APIs [43]. Moreover, these findings are in accordance with those of Yabre *et al*. who achieved 100% specificity with correct discrimination of falsified samples [5,44].

As one can see, the results obtained with NIR spectroscopy were confirmed by HPLC analysis in terms of API identification since their detection was positive for all the samples.

However, none of the substandard samples detected by HPLC tests were detected with the OCC models. These substandard samples had contents in the range: 72–88% of the label claimed [29]. It is important to notice that the samples involved in this study are high dose solid pharmaceutical forms, and the OCC models were not designed to detect small variations of API content. Indeed, the variability between the different formulations (e.g. use of different pharmaceutical excipients) was higher than the variability due to quantitative issues. In order to detect substandard formulations, it is recommended to build models specific to a formulation or a group of similar formulations.

These results are close to those obtained by Zambrzycki *et al*. who found that only 19% of the 80% API substandard and 48% of the 50% API substandard were detected with an NIR-SG-1 [27]. However, Wang *et al*. were able to detect either substandard or falsified samples with a handheld NIR-S-G-1 device using quantitative models [45].

The implementation of NIR-device has some drawbacks. On the one hand, before analyses, there is the lamp pre-heating phase, the reference (99% reflectance standard spectralon®) measurement, and tablet removal from blister packs, (especially with aluminum-aluminum ones) which increases the analysis time. These constraints make the use of NIR-device less suitable during inspection phase. This pre-heating and reference measurement may be avoided using the same reference for each sample and removing the highly variable spectral ranges. However, the performances of the subsequent models may be affected by these shortcuts (not tested in this study). The NIR spectra are not directly interpretable and chemometric tools are required [42,46]. Moreover, the spectral database has to be as representative and up-to-date as possible. In the same way, it would be necessary to foresee the transfer of spectra between devices since differences between devices may lead to variations in the spectra [47]. These tasks are time-consuming and require well-trained staff before implementing the tools in the field.

### Paper Analytical Devices (PADs)

#### Results from visual inspection of the PADs

PADs were used only to analyze ciprofloxacin samples. All the 150 tested samples were positive for API identification by visual inspection (blue color on Lane D and orange color on Lane L) leading to a sensitivity of 100% (S4 Table). The picture of each sample’s PAD was compared to a reference PAD run with a pure sample of ciprofloxacin. Since the interpretation was done with the naked eye, it is subjective. The cards were interpreted and the results were confirmed by experienced PAD users at the University of Notre Dame. The observed sensitivity was consistent with those of other studies where PADs were implemented [24,27,37,48]. As expected, the six substandard samples that failed HPLC assay were not detected by PADs. These results are similar to those obtained in Malawi [24] and Laos [27,28].

**Fig 5:**
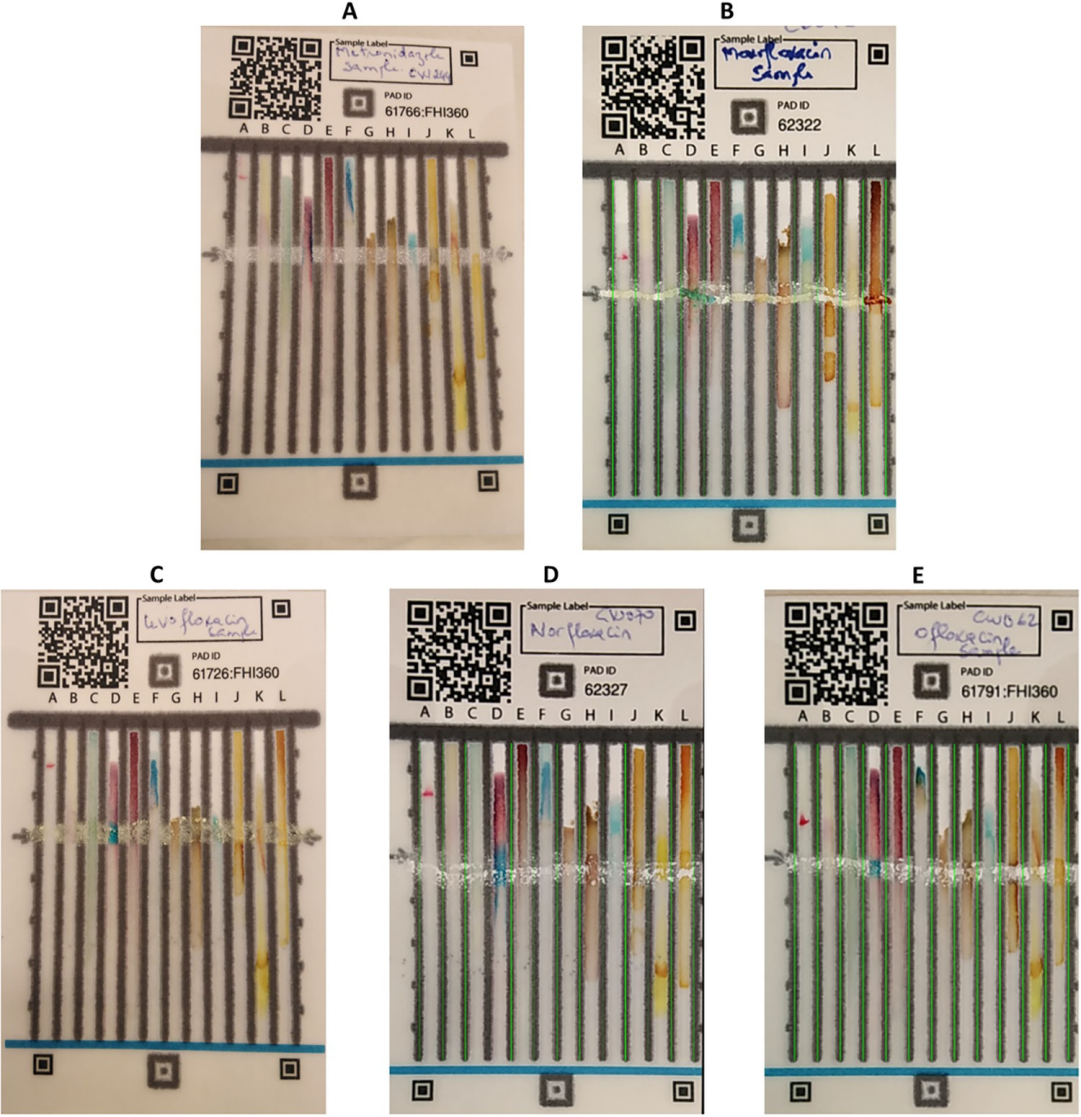
Cards of samples tested for specificity determination. A) testing of a metronidazole sample; B) testing of moxifloxacin sample; C) testing of levofloxacin sample; D) testing of norfloxacin sample; E) testing of ofloxacin sample. Lanes D and L were positive with blue and orange colors respectively for cards C (Levofloxacin sample), D (Norfloxacin samples), and E (Ofloxacin samples). The coloration of lane D for card B (Moxifloxacin) tends to green color.

Samples containing APIs belonging to the fluoroquinolone family: ofloxacin, levofloxacin, norfloxacin and moxifloxacin (S2 Table) were also tested with ciprofloxacin cards before moving to the field. These tests showed that the cards were not specific to ciprofloxacin, since the D and L lanes gave blue and orange colorations respectively for these substances except for moxifloxacin, for which the color obtained on lane D tended to greenish-blue (Fig 5A–5E). These results can be considered as false positive ones. Lane D detects tertiary amines, while lane L detects 1, 3-dicarbonyls. These two functional groups are present in all the APIs of the fluoroquinolone family. Thus, when used by a knowledgeable operator, the PAD can narrow down the possible chemical class of an unknown API in a field setting. The specificity of PADs for this study was 40.0% although the number of tested samples different from ciprofloxacin was not representative (S4 Table). For screening devices, Zambrzycki *et al*. [27] recommend to focus on sensitivity rather than specificity regarding potential public health risks that may occur.

#### Results from use of the PADreader phone app

The photos of the PADs for 150 samples of ciprofloxacin were evaluated with an Android phone app, PADreader [38]. Additional information about the PADreader are given in S2 File. Despite some potential problems (See S2 File), the PADreader was able to capture 149 of the 150 PAD images from a computer monitor or laptop screen, and all of these images were correctly classified as ciprofloxacin. One image was photographed in poor focus, and the PADreader could not find its fiducial marks; this card could not be read by the app. The samples containing APIs belonging to fluoroquinolones family were all read as ciprofloxacin by the app. In contrast to the app, the visual interpretation of the PADs results was able to distinguish moxifloxacin thanks to the greenish-blue color instead of blue on lane D. The neural net classifier of the PADreader would likely be able to distinguish moxifloxacin if it has been trained before with this substance.

#### Observations in the implementation of PADs

Good performances were obtained regarding sensitivity either with visual inspection or with the PADreader as no false negatives and no cases of substitution of API were found within the 150 samples of ciprofloxacin tested. These results were confirmed by HPLC tests [29]. In contrast to the results obtained by Caillet *et al*., [26] no misclassification problems with PADs occurred in this study.

The presence of starch was detected by the tri-iodide reagent in lane J of the PAD in many of the samples. Some tablet formulations approved by the relevant regulatory authority include starch as a tableting agent, and starch would be an acceptable component in these formulations. Other tablet formulations use different tableting agents, such as microcrystalline cellulose, so observation of starch in these formulations would indicate that the manufacturer was not using the approved formulation. Unfortunately, the approved compositions of most of the formulations were not available, limiting the utility of this additional information.

Some drawbacks attributed to PADs are firstly, their shelf life, which depends on storage conditions. When contained in their sealed packaging, they remain stable for at least one year when stored in a refrigerator or at least four months under tropical conditions [49]. The need for refrigerated storage is a limiting factor for their use in developing countries since the availability of electrical power and logistical facilities are not always guaranteed. Moreover, four months is somewhat short considering logistical problems encountered in developing countries [12]. Secondly, the 12-lane PAD used in this study can only detect a limited range of 20 APIs, which limits their versality. Thirdly, some sample preparation is necessary, consisting of crushing the tablet before analysis. Therefore they cannot be easily implemented during an inspection in a pharmacy, for example.

### Experience and relevance of using screening tools

Considering the protocol of the sampling strategy (covert strategy, using mystery shoppers) used during the prevalence study [29], the samples were not tested directly in the pharmacies. They were transported to a different location and were tested all together. It is important to note that laboratory facilities were not needed for their implementation. A constraint in the implementation of each tested screening device concerned the tablets in aluminum/aluminum blister pack. This packaging prevents the correct application of visual inspection of the tablets and any testing by NIR or PADs. These samples had to be removed from their packaging which may affect their stability.

Concerning the simplified visual inspection checklist (S6 Fig), the analysis time was estimated to three minutes per sample as questions were only related to solid dosage forms.

In remote areas of resource-limited settings for example, concerning the final decision making for samples with status ‘C’ (samples with visual non-conformities that can certainly compromise their safety or efficacy), there are not always qualified staff such as pharmacists or medical doctors who would be able to evaluate the benefit/risk and decide what to do with such medicines as advised by Schiavetti *et al*. [15]. In such situations it would be preferable to adopt a precautionary approach and to quarantine the suspect product–while issuing a complaint to the supplier and informing the local health authority about it. Nevertheless, our experience confirmed that the simplified visual inspection checklist is a user-friendly tool that is easy to implement. It can be used by non-specialists and does not require, as opposed to other tools for visual inspection [50,51], the access to additional information related to pharmaceutical regulation. It is an affordable and easy to implement tool that can detect some SF medicines before they reach the patient. Furthermore, it can empower the field health staff and increase their awareness of the risks related to SF medicines. Its systematic use can strengthen pharmaceutical regulatory and medicines quality assurance systems. Visual inspection is particularly urgent in many resource-limited settings [15,41], but it would be beneficial even in settings where SF medicines are rare, but never completely absent. In addition, visual inspection should always be performed to avoid overconfidence of inspectors in other devices (e.g. NIR spectrophotometer) as observed by Caillet *et al*. [25]. This overconfidence may sometimes lead to acceptance of samples despite there being evident failures observable on the packaging.

The NIR device was connected to a computer with a universal serial bus (USB) during the analysis. It can also be operated with cell phones or tablets via a wireless Bluetooth connection and a dedicated smartphone application. The recording of a spectrum from a single tablet was completed in less than 60 seconds. For each sample, it has been preferred to increase the number of tablets measured rather than repeating measurements on the same tablet. The choice of 10 tablets measured per sample is based on the fact that most blisters found on the African pharmaceutical market contain 10 tablets. Moreover, in the context of medicines falsification, it is hardly possible to be sure that the composition of tablets is similar for all tablets of the same blister. Therefore, analyzing all tablets of the blister enables to get a more representative dataset since the “inter-tablet” variability is better assessed. The resulting spectral data subsequently had to be in imported into the chemometric software, pretreated, and analyzed. Tablets were removed from the blister packaging before analysis because some samples were contained in aluminum-aluminum blisters, which are impervious to NIR wavelengths. We were also concerned that the plastic in PVC-Aluminum blisters could have been damaged by exposure to the sun (UV light), for example for samples collected in the informal sector, without necessarily impacting the product quality. This might impact the NIR data analytics and result in unfair rejection of a product because of problems related to the blister. However, this operating mode make the implementation of NIR unsuitable during inspection phase. In the present study, the interpretation of NIR results was achieved using the OCC models developed and validated. Even though the tested NIR device may be operated by non-spectroscopists, chemometric models used to evaluate test results require ongoing work by skilled personnel. In particular, the models require reliable, up-to-date and exhaustive libraries of NIR spectra that include multiple batches of each quality assured formulation and brand, and continual testing of the data analytics algorithms as new spectra enter the library. Although encouraging results were obtained with ciprofloxacin and metronidazole OCC models, their calibration data sets need to be updated with more formulations and brands [52]. In addition, according to the findings obtained with the OCC models, it is more important that the calibration set reflects the brands that are in a particular market, than that it includes many brands that are not in the market. Therefore, it is very important to check the library composition for a chemometric model and to ensure that the test samples belong to this domain before trusting the model’s predictions. Since many portable spectrophotometers use proprietary chemometric models, this information may not always be available to the user. Although the spectra used to build the OCC models were acquired in Belgium and the test spectra were collected in Cameroon, good performance was obtained for API identification. In Cameroon, temperatures and relative humidity were both higher than those observed in Belgium. However, these environmental parameters, which usually affect handheld NIR spectra, had negligible influence on the obtained results as long as the lamp was left ON before analysis of samples in the field until the relative humidity dropped below 10%. In some cities the heating time of the lamp was longer than in others because of higher relative humidity.

The PAD is an easy to make screening tool which can be produced by students as demonstrated in the paper [37]. In contrast to NIR spectroscopy or visual inspection which can be used on multiple samples without sample preparation, it is a single use device that requires crushing the tablet to powder. In addition, PADs are not available for all APIs. For example, at the time of this study, metronidazole could not be analyzed on the PAD. The analysis time with PADs was greater than the other screening tools, approximately 7 minutes per sample from crushing to color code reading. However, several analyses can be run at the same time decreasing the total analysis time per sample.

Overall, all of these screening tools are fast and user-friendly in comparison to conventional methods such as TLC, and can be used by non-specialized staff with little training. They are eco-friendly as well-NIR and the simplified visual inspection checklist do not require any sample preparation and PADs do not require any organic solvent for elution. Furthermore, they are inexpensive. The simplified visual inspection checklist is free of charge, and can potentially be adapted for different formulations. The price of a PAD card was originally estimated at USD$3 [25,26,48] and they are now available for USD$2 [53]. The handheld NIR system used in this study costs about USD$1600 (Table 1).

The implementation of screening tools would depend on the objectives and the available resources (financial and human). They should be used in a complementary way. For example, the visual inspection checklist can be used to sort suspicious samples with visual non-conformities in a first stage for on-site or near-site analysis during post-marketing monitoring operations and/or field studies. Most authors emphasize that it is important to systematically perform visual inspection in a first stage before applying other screening tools [25,41]. For example, in this study, samples that had to be quarantined due to suspicious appearance of tablets or lack of critical information on the packaging materials, could be swiftly discarded as the risks are greater than benefits for the patients. Then, a system optimized for API identification like PADs or handheld NIR devices with OCC models could be applied in a screening phase in post-marketing surveillance operations and/or field studies to reveal falsification problems which cannot be detected with the naked eye such as lack or substitution of API. It is true that during this study such situation was not encountered, but substances with chemical structures close to the target API and placebo were used to mimic possible falsifications For this stage, spectroscopic systems like NIR handheld devices are highly recommended [54], since they can help to reduce the number of routine analyses in a first stage, saving time and money. Moreover, they leave the samples undamaged, allowing further tests. Therefore, only the samples failing to this screening phase and those randomly selected would be analysed during a confirmatory phase with compendial methods.

Digitization could be considered for the simplified checklist as emphazised by Schiavetti *et al*. [15], and smartphone applications should be developped for NIR handheld device models in order to improve the speed of getting results. This approach has been developed, validated and applied by some authors in the frame of field detection of illicit drugs [55]. A smartphone application is already available for PAD image analysis.

In Cameroon where this study was implemented, the department of pharmacy, drugs and laboratories (DPML), in conjunction with the national drug and valuation laboratory (LANACOME), is responsible for the verification of the quality of medicines manufactured or used in the country. The monitoring of the pharmaceutical market is the responsibility of the general inspectorate of pharmaceutical services and laboratories (IGSPL). It coordinates the fight against the illegal sale of medicines and SF medicines. It should be noted that the post-marketing inspection of the pharmaceutical services is not very regular due to a lack of logistical and financial resources [56]. Considering these difficulties usually encountered by under-resourced regulators, screening tools may clearly be helpful.

These types of screening tools would enable national regulatory authorities and other stakeholders like manufacturers in their post-marketing surveillance, health care providers, pharmacist, pharmaceutical wholesalers and others to conduct post-market surveillance activities and react quickly in response to reports about suspicious medicines. These suspicious samples would be quarantined before full confirmatory analysis are run at an accredited QC laboratory, which in most cases are necessary in order to take some final decision about suspect products. The health of the population would therefore be preserved in case of proven non-compliance or falsification. These screening tools could be very useful especially in hard-to-reach rural areas that are not always covered by medicine quality monitoring programs. It is therefore important to emphasize that the choice of the screening tool depends on the goal to be achieved, and that the results of the screening must be interpreted by qualified staff who know well their strengths and weaknesses [43,57,58].

## Conclusion

The usefulness, drawbacks, and risks of screening tools in the frame of post-marketing surveillance and particularly in prevalence studies has been discussed along this paper. The choice of one and/or other tools will depend on the available resources and the intended purpose. The visual inspection checklist used in this study revealed mainly issues related to the quality of the packaging, the (lack of) traceability, and possible accelerated deterioration of the products. However, it would be important to improve the simplified checklist in the sense of assigning status ‘C’ to medicinal products lacking information about the manufacturing company on both the primary and secondary packaging. As mentioned by Schiavetti *et al*., the checklist can be modelled according to needs of different countries [15]. The physicochemical screening techniques showed overall good performances. Using PADs, all ciprofloxacin samples were correctly identified as such, but they could not be separated from other fluoroquinolones. However, NIR spectroscopy could distinguish tablets containing these different APIs. In addition to that, a significant spectral library was acquired for ciprofloxacin and metronidazole tablets during this study. Nevertheless, the need for updating spectral database is emphasized as it has to be representative of the samples present on the pharmaceutical market. Each national regulatory authority should supervise the development of a library of spectra of quality-assured medicines in order to obtain a more reliable and exhaustive database [54]. Library construction may also be envisaged at a higher level, such as the regional level in case of regulatory harmonization initiatives, or at the WHO, although the proliferation of both portable spectrophotometers and medicine manufacturers, brands, and batches make this a challenging task. The main advantage of NIR over PADs is its versatility, enabling the detection of APIs or specific formulations as well as the quantitative analysis of samples. However, the main drawback is that the performances of these applications are dependent on the database and the building and maintenance of chemometric models.

The main limitation of this study is the fact that the samples were mostly compliant regarding chemical identification (no absence or change of API). A different situation such as the absence of API would have permitted an objective evaluation of the specificity of the different tools. Nevertheless, national health policies and regulatory agencies in resource-limited contexts should invest in these types of cheap and easy-to-implement methods, which could strengthen the quality monitoring of medicines particularly for post-maketing surveillance in remote areas. The present work could be extended to other essential medicines and other pharmaceutical forms, especially in areas with limited or absent post-marketing surveillance activities.

## Supporting information

S1 Fig Suitability test on ciprofloxacin card (PAD). A) Card run without sample. B) Card run with Ciprofloxacin reference standard. C) Card run with a sample of ciprofloxacin. Lanes D and L were positive with blue and orange colors respectively for cards B and C.
(TIF)

S2 Fig Pictures showing some samples with visual non-conformities. A) Sample with internal packaging cut in two parts; B) Sample with lack of information about manufacturing laboratory either on primary or secondary packaging; C) Sample with a half tablet in an intact blister; D) Sample with tablets having stains.
(TIF)

S3 Fig Extreme plots of DD-SIMCA models. A) extreme plot of Ciprofloxacin *a priori* model; B) Extreme plot of Ciprofloxacin *a posteriori* model; C) Extreme plot of Metronidazole *a priori* model; D) Extreme plot of Metronidazole *a posteriori* model. The red dots represent samples of the calibration set and the blue vertical lines delimit the tolerance area.
(TIF)

S4 Fig PCA plots on ciprofloxacin samples spectra. A represents PC1 vs PC2, PC1 vs PC3 and PC2 vs PC3 plots on spectra collected in Belgium (red) and Cameroon (green). B represents PC1 vs PC2, PC1 vs PC3 and PC2 vs PC3 on a posteriori training (red) and test sets (green) obtained with Kennard-Stone algorithm.
(TIF)

S5 Fig PCA plots on metronidazole samples spectra. A represents PC1 vs PC2, PC1 vs PC3 and PC2 vs PC3 plots on spectra collected in Belgium (red) and Cameroon (green). B represents PC1 vs PC2, PC1 vs PC3 and PC2 vs PC3 on *a posteriori* training (red) and test (green) sets (green) obtained with Kennard-Stone algorithm.
(TIF)

S6 Fig Simplified Visual inspection checklist. (TIF)

S1 Table List of ciprofloxacin and metronidazole samples collected for the construction of DD-SIMCA models (calibration and validation sets). (DOCX)

S2 Table List of samples used for the specificity evaluation of DD-SIMCA models and PADs. (DOCX)

S3 Table Test set samples concerned about outliers with initial OCC models. (DOCX)

S4 Table Confusion matrix of Ciprofloxacin PADs results. (DOCX)

S1 File NIR supplementary data. (DOCX)

S2 File PADreader supplementary information. (DOCX)

## References

[1] Green MD, Hostetler DM, Nettey H, Swamidoss I, Ranieri N, Newton PN. Integration of Novel Low-Cost Colorimetric, Laser Photometric, and Visual Fluorescent Techniques for Rapid Identification of Falsified Medicines in Resource-Poor Areas: Application to Artemether–Lumefantrine. Am J Trop Med Hyg. 2015;92 Suppl 6:8–16. Available at doi: 10.4269/ajtmh.14-0832 25897066 PMC4455085

[2] WHO 2017, A Study on the Public Health and Socioeconomic Impact of Substandard and Falsified Medical Products. Available from: https://www.who.int/publications/i/item/9789241513432. Accessed October 25, 2021.

[3] Guzman J, O’Connell E, Kikule K, Hafner T. The WHO Global Benchmarking Tool: a game changer for strengthening national regulatory capacity. *BMJ Glob Health*. 2020;5, e003181. Available at: doi: 10.1136/bmjgh-2020-003181 32784212 PMC7418656

[4] Macé C, Rägo L, Ravinetto R. How the concept of WHO-listed authorities will change international procurement policies for medicines. *BMJ Global Health* 2022;6:e008109. Available at: doi: 10.1136/bmjgh-2021-008109 35144982 PMC8883025

[5] Yabré M, Ferey L, Sakira AK, Bonmatin C, Fauré C, Somé TI, Gaudin K. Green analytical methods of antimalarial artemether-lumefantrine analysis for falsification detection using a low-cost handled NIR spectrometer with DD-SIMCA and drug quantification by HPLC. Molecules. 2020;25(15). Available at: doi: 10.3390/molecules25153397 32727052 PMC7435840

[6] Vickers S, Bernier M, Zambrzycki S, Fernandez FM, Newton PN, Caillet C. Field detection devices for screening the quality of medicines: a systematic review. BMJ Glob Heal. 2018;3:725. Available at: doi: 10.1136/bmjgh-2018-000725 30233826 PMC6135480

[7] Kovacs S, Hawes SE, Maley SN, Mosites E, Wong L, Stergachis A. Technologies for Detecting Falsified and Substandard Drugs in Low and Middle-Income Countries. PLoS One. 2014;9(3):11. Available at: doi: 10.1371/journal.pone.0090601 24671033 PMC3966738

[8] Bakker-’t Hart IME, Ohana D, Venhuis BJ. Current challenges in the detection and analysis of falsified medicines. J Pharm Biomed Anal. 2021;197:113948. Available at: doi: 10.1016/j.jpba.2021.113948 33582458

[9] WHO Africa. "Tanzania is first African country to reach an important milestone in the regulation of medicines". Available from: https://www.afro.who.int/news/tanzania-first-african-country-reach-important-milestone-regulation-medicines Accessed February 06^th^ 2023.

[10] Tobolkina E, Rudaz S. Capillary Electrophoresis Instruments for Medical Applications and Falsified Drug Analysis/Quality Control in Developing Countries. Anal. Chem. 2021;93(23):8107–8115. Available at: doi: 10.1021/acs.analchem.1c00839 34061489

[11] Rahman MS, Yoshida N, Tsuboi H, Tomizu N, Endo J, Miyu O, et al. The health consequences of falsified medicines- A study of the published literature. Trop Med Int Heal. 2018;23(12):1294–303. Available at: doi: 10.1111/tmi.13161 30291683

[12] Höllein L, Kaale E, Mwalwisi YH, Schulze MH, Holzgrabe U. Routine quality control of medicines in developing countries: Analytical challenges, regulatory infrastructures and the prevalence of counterfeit medicines in Tanzania. TrAC. 2016;76:60–70. Available at: 10.1016/j.trac.2015.11.009.

[13] Roth L, Nalim A, Turesson B, Krech L. Global landscape assessment of screening technologies for medicine quality assurance: stakeholder perceptions and practices from ten countries. Glob. Health. 2018;14:43. Available at: doi: 10.1186/s12992-018-0360-y 29695278 PMC5922304

[14] Roth L, Biggs KB, Bempong DK. Substandard and falsified medicine screening technologies. AAPS Open. 2019;5:2. Available at: 10.1186/s41120-019-0031-y.

[15] Schiavetti B, Wynendaele E, Melotte V, Van Der Elst J, De Spiegeleer B, Ravinetto R. A simplified checklist for the visual inspection of finished pharmaceutical products: A way to empower frontline health workers in the fight against poor-quality medicines. J Pharm Policy Pract. 2020;13(1). Available at: doi: 10.1186/s40545-020-00211-9 32377348 PMC7193355

[16] Waffo Tchounga CA, Sacre PY, Ciza P, Ngono R, Ziemons E, Hubert P, et al. Composition analysis of falsified chloroquine phosphate samples seized during the COVID-19 pandemic. J Pharm Biomed Anal. 2021;194(5):113761. Available at: doi: 10.1016/j.jpba.2020.113761 33234414 PMC7659915

[17] Sacré P-Y, Deconinck E, De Beer T, Courselle P, Vancauwenberghe R, Chiap P, et al. Comparison and combination of spectroscopic techniques for the detection of counterfeit medicines. J Pharm Biomed Anal. 2010;53:445–53. Available at: doi: 10.1016/j.jpba.2010.05.012 20542652

[18] Schiavetti B, Wynendaele E, De Spiegeleer B, Mbinze GJ, Eme Kalenda N, Marini R, et al. The Quality of Medicines Used in Children and Supplied by Private Pharmaceutical Wholesalers in Kinshasa, Democratic Republic of Congo: A Prospective Survey. Am J Trop Med Hyg. 2018;98(3):894–903. Available at: doi: 10.4269/ajtmh.17-0732 29313479 PMC5930909

[19] Khuluza F, Kigera S, Heide L. Low Prevalence of Substandard and Falsified Antimalarial and Antibiotic Medicines in Public and Faith-Based Health Facilities of Southern Malawi. Am J Trop Med Hyg. 2017;96(5):1124–35. Available at: doi: 10.4269/ajtmh.16-1008 28219993 PMC5417205

[20] Weaver AA, Reiser H, Barstis T, Benvenuti M, Ghosh D, Hunckler M, et al. Paper analytical devices for fast field screening of beta lactam antibiotics and anti-tuberculosis pharmaceuticals. Anal Chem. 2013;85(13):6453–60. Available at: doi: 10.1021/ac400989p 23725012 PMC3800146

[21] World Health Organization, Survey of the Quality of Selected Antimalarial Medicines Circulating in Six Countries of Sub-Saharan Africa; 2011. Available at: https://www.afro.who.int/sites/default/files/2017-06/WHO_QAMSA_report.pdf. Accessed October 25, 2021.

[22] Frimpong-Manso Opuni K, Nettey H, Larbi MA, Naa S, Amartey A, Nti G, et al. Usefulness of combined screening methods for rapid detection of falsified and/ or substandard medicines in the absence of a confirmatory method. Malar J. 2019;18:403. Available at: doi: 10.1186/s12936-019-3045-y 31805937 PMC6896689

[23] Petersen A, Held N, Heide L. Surveillance for falsified and substandard medicines in Africa and Asia by local organizations using the low-cost GPHF Minilab. PLoS One 2017;12: e0184165. Available at: doi: 10.1371/journal.pone.0184165 28877208 PMC5587284

[24] Chikowe I, Bliese SL, Lucas S, Lieberman M. Amoxicillin Quality and Selling Practices in Urban Pharmacies and Drug Stores of Blantyre, Malawi. Am J Trop Med Hyg. 2018;99(1):233–8. Available at: doi: 10.4269/ajtmh.18-0003 29692302 PMC6085786

[25] Caillet C, Vickers S, Vidhamal V, Boutsamay K, Boupha P, Zambrzycki S, et al. Evaluation of portable devices for medicine quality screening: Lessons learnt, recommendations for implementation, and future priorities. PLoS Med. 2021;18(9), e1003747. Available at: doi: 10.1371/journal.pmed.1003747 34591861 PMC8483386

[26] Caillet C, Vickers S, Zambrzycki S, Fernández FM, Vidhamaly V, Boutsamay K, et al. A comparative field evaluation of six medicine quality screening devices in Laos. PLoS Negl Trop Dis. 2021;15(9):e0009674. Available at: doi: 10.1371/journal.pntd.0009674 34591852 PMC8483322

[27] Zambrzycki SC, Caillet C, Vickers S, Bouza M, Donndelinger DV, Geben LC, et al. Laboratory evaluation of twelve portable devices for medicine quality screening. PLoS Negl Trop Dis. 2021;15(9):e0009360. Available at: doi: 10.1371/journal.pntd.0009360 34591844 PMC8483346

[28] Luangasanatip N, Khonputsa P, Caillet C, Vickers S, Zambrzycki S, Fernández FM, et al. Implementation of field detection devices for antimalarial quality screening in lao pdr—a cost-effectiveness analysis. PLoS Negl Trop Dis. 2021;15(9):1–19. Available at: doi: 10.1371/journal.pntd.0009539 34591842 PMC8483304

[29] Waffo Tchounga CA, Sacre PY, Ciza PH, Ngono R, De Bleye C, Ziemons E, et al. Prevalence of poor-quality ciprofloxacin and metronidazole tablets in three cities of Cameroon. Am J Trop Med Hyg. 2022;108(2):403–411. Available at: 10.4269/ajtmh.22-0221. 36535257 PMC9896317

[30] Schäfermann S, Neci R, Ngah Ndze E, Nyaah F, Basolanduma Pondo V, Heide LI. Availability, prices and affordability of selected antibiotics and medicines against non-communicable diseases in western Cameroon and northeast DR Congo. PLoS ONE 2020;15(1): e0227515. Available at: doi: 10.1371/journal.pone.0227515 31910444 PMC6946586

[31] Coic L, Sacré P-Y, Dispas A, Karim Sakira A, Fillet M, Marini RD, et al. Comparison of hyperspectral imaging techniques for the elucidation of falsified medicines composition. Talanta. 2019;198:457–463. Available at: doi: 10.1016/j.talanta.2019.02.032 30876587

[32] Zontov YV, Rodionova OY, Kucheryavskiy S V, Pomerantsev AL. DD-SIMCA–A MATLAB GUI tool for data driven SIMCA approach. Chemom Intell Lab Syst. 2017;167:23–8. Available at: 10.1016/j.chemolab.2017.05.010.

[33] Kennard RW, Stone LA. Computer Aided Design of Experiments. Technometrics. 1969;11(1):137–48. Available at: 10.2307/1266770.

[34] Kennardstone—Eigenvector Research Documentation Wiki. Available from: https://wiki.eigenvector.com/index.php?title=Kennardstone Accessed on 28^th^ July 2022.

[35] Milman BL. Reliability and Errors of Identification. In: Chemical Identification and its Quality Assurance. Springer, Berlin, Heidelberg; 2011. pp63–113 Available from: 10.1007/978-3-642-15361-7_4 Accessed on December 21^th^ 2021.

[36] Weaver AA, Lieberman M. Paper test cards for presumptive testing of very low quality antimalarial medications. Am J Trop Med Hyg. 2015;92(6):17–23. Available at: doi: 10.4269/ajtmh.14-0384 25897064 PMC4455083

[37] Bliese SL O’Donnell D, Weaver AA, Lieberman M. Paper Millifluidics Lab: Using a Library of Color Tests to Find Adulterated Antibiotics. J Chem Educ. 2020 10;97(3):786–92. Available at: 10.1021/acs.jchemed.9b00433. 32174646 PMC7066646

[38] Bannerjee S, Sweet J, Lieberman M, Flynn P, and Sweet C.Visual Recognition of Paper Analytical Device Images for Detection of Falsified Pharmaceuticals. Accepted for WACV 2016: IEEE Winter Conference on Applications of Computer Vision, March 7–9, Lake Placid NY. Available at: https://ieeexplore.ieee.org/document/7477598 Accessed June 02nd 2023.

[39] Essomba N, Adiogo D, Essome MJ, Lehman L, Coppieters Y. Habitudes d’approvisionnement en médicaments par les populations d’une ville semi-rurale au Cameroun. Health Sci. Dis. 2014;15:1–7. Available at: https://www.hsd-fmsb.org/index.php/hsd/article/view/438.

[40] WHO Technical Report Series 902 WHO expert committee on specifications for pharmaceutical preparations.World Health Organ Tech Rep Ser. thirty-sixth report. Geneva 2002. Available from: http://apps.who.int/iris/bitstream/handle/10665/42424/WHO_TRS_902.pdf;jsessionid=6BFB0F6F9214072FBA3BC7937C78F1D6?sequence=1 Accessed on September 20^th^ 2021.

[41] Mohamed Ali GK, Ravinetto R, Alfadl AA. The importance of visual inspection in national quality assurance systems for medicines. J of Pharm Policy and Pract 2020;13(52): 1–3. Available at: 10.1186/s40545-020-00264-w. 32939287 PMC7487550

[42] Tack B, Vita D, Ntangu E, Ngina J, Lunguya O, Vangeluwe D, et al. Poor availability of age-appropriate drug formulations in DR Congo: a barrier to switch from intravenous to oral antibiotics in children admitted to Kisantu hospital. Abstract presented at ESDPPP, Liverpool 2022. Available at: https://www.esdppp.org/app/download/11171937152/ESDPPP+Congress+Programme.pdf?t=1657009224 Accessed on October, 23th 2022.

[43] Ciza PH, Sacre P-Y, Waffo C, Coïc L, Avohou H, Mbinze JK, et al. Comparing the qualitative performances of handheld NIR and Raman spectrophotometers for the detection of falsified pharmaceutical products. Talanta. 2019;202:469–78. Available at: doi: 10.1016/j.talanta.2019.04.049 31171209

[44] Yabré M, Sakira AK, Bandé M, Goumbri BWF, Ouattara SM, Fofana S, et al. Detection of Falsified Antimalarial Sulfadoxine-Pyrimethamine and Dihydroartemisinin-Piperaquine Drugs Using a Low-Cost Handheld Near-Infrared Spectrometer. JAMC. 2022; 5335936; Available at: doi: 10.1155/2022/5335936 35558651 PMC9090531

[45] Wang W, Keller MD, Baughman T, Wilson BK. Evaluating Low-Cost Optical Spectrometers for the Detection of Simulated Substandard and Falsified Medicines. Appl Spectrosc. 2020;74(3):323–333 Available at: doi: 10.1177/0003702819877422 31617368 PMC7066480

[46] Deidda R, Sacre P-Y, Clavaud M, Coïc L, Avohou H, Hubert P, et al. Vibrational spectroscopy in analysis of pharmaceuticals: Critical review of innovative portable and handheld NIR and Raman spectrophotometers. TrAC. 2019;114:251–9. Available at: 10.1016/j.trac.2019.02.035.

[47] Ciza PH, Sacre P-Y, Waffo C, Kimbeni TM, Masereel B, Hubert P, et al. Comparison of several strategies for the deployment of a multivariate regression model on several handheld NIR instruments. Application to the quality control of medicines. J Pharm Biomed Anal. 2022;215:114755. Available at: doi: 10.1016/j.jpba.2022.114755 35430411

[48] Chen HH, Higgins C, Laing SK, Bliese SL, Lieberman M, Ozawa S. Cost savings of paper analytical devices (PADs) to detect substandard and falsified antibiotics: Kenya case study. Med Access Point Care. 2021;5:239920262098030. Available at: doi: 10.1177/2399202620980303 33834120 PMC8026160

[49] USP Technology Review: Paper Analytical Device (PAD). Available at: https://padproject.nd.edu/assets/422967/usp_paper_analytical_device_pad.pdf Accessed on September 12th 2022.

[50] World Health Organization: Be Aware tool for visual inspection of medicines. A checklist for visual inspection of medicines in order to identify suspicious products for further examination. (2018) Available at: https://www.whpa.org/sites/default/files/2018-12/Toolkit_BeAware_Inspection.pdf Accessed March 30th 2023.

[51] Inspection visuelle des médicaments. Liste de contrôle pour l’inspection visuelle des médicaments et le dépistage des produits suspects. Available at: https://www.fip.org/files/fip/counterfeit/VisualInspection/A%20tool%20for%20visual%20inspection%20of%20medicines%20FR.pdf Accessed March 30th 2023.

[52] Rodionova OY, Pomerantsev AL. NIR-based approach to counterfeit-drug detection. Trends Anal Chem. 2010;29:795–803. Available at: 10.1016/j.trac.2010.05.004.

[53] https://paperanalytics.org/products/paper-analytical-device-pad-20-pack?variant=39956974796989. Accessed on November, 07th 2022.

[54] Wilson BK, Kaur H, Allan EL, Lozama A, and Bell D. A New Handheld Device for the Detection of Falsified Medicines: Demonstration on Falsified Artemisinin-Based Therapies from the Field. Am J Trop Med Hyg. 2017;96(5):1117–23. Available at: doi: 10.4269/ajtmh.16-0904 28219992 PMC5417204

[55] Coppey F, Bécue A, Sacré P-Y, Ziemons EM, Hubert P, Esseiva P. Providing illicit drugs results in five seconds using ultra-portable NIR technology: An opportunity for forensic laboratories to cope with the trend toward the decentralization of forensic capabilities. Forensic Science International. 2020;317:110498; Available at: doi: 10.1016/j.forsciint.2020.110498 33017781

[56] Politique Pharmaceutique Nationale Du Cameroun (PPN) - 2013. https://dpml.cm/index.php/fr/s-informer/actualite/annee-2018/546-politique-pharmaceutique-nationale-du-cameroun-ppn-2013. Accessed on October 08^th^ 2021.

[57] Jähnke R. Letter to the Editor on Previously Published GPHF-Minilab Assessment. Am J Trop Med Hyg. 2018;98(6):1880. Available at: doi: 10.4269/ajtmh.18-0127a 29877172 PMC6086185

[58] Noviana E, Carrão DB, Pratiwi R, Henry CS. Emerging applications of paper-based analytical devices for drug analysis: A review. Anal Chim Acta. 2020;1116:70–90. Available at: doi: 10.1016/j.aca.2020.03.013 32389191

