# Supplementary figures and images for: Usefulness of medicine screening tools in the frame of pharmaceutical post-marketing surveillance
Source: PLoS One. 2023 Aug 11;18(8):e0289865. doi: 10.1371/journal.pone.0289865 (PMC10420354; doi:10.1371/journal.pone.0289865)

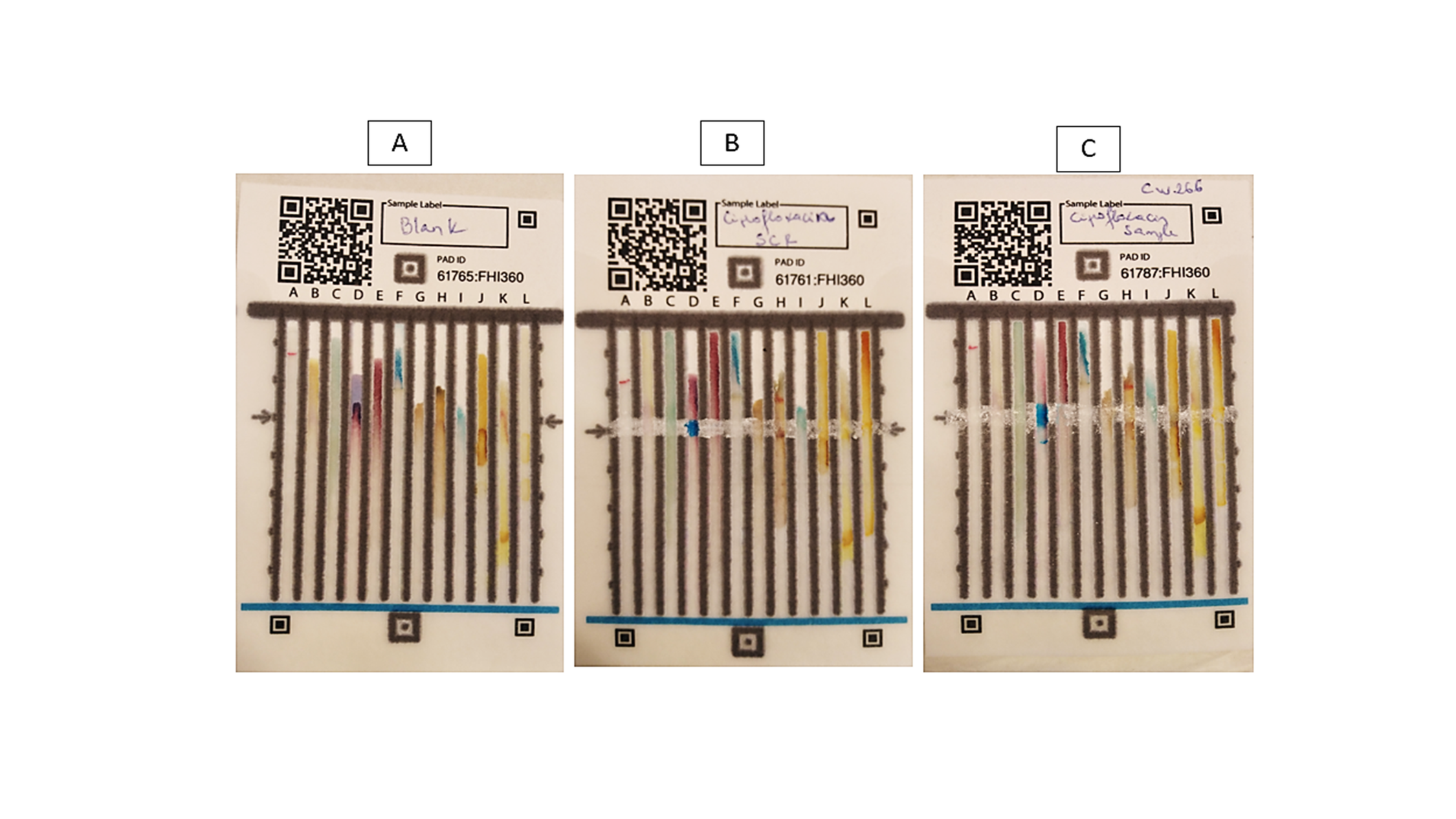

Supplement: S1 Fig — A) Card run without sample. B) Card run with Ciprofloxacin reference standard. C) Card run with a sample of ciprofloxacin. Lanes D and L were positive with blue and orange colors respectively for cards B and C. (TIF) [file pone.0289865.s001.tif]

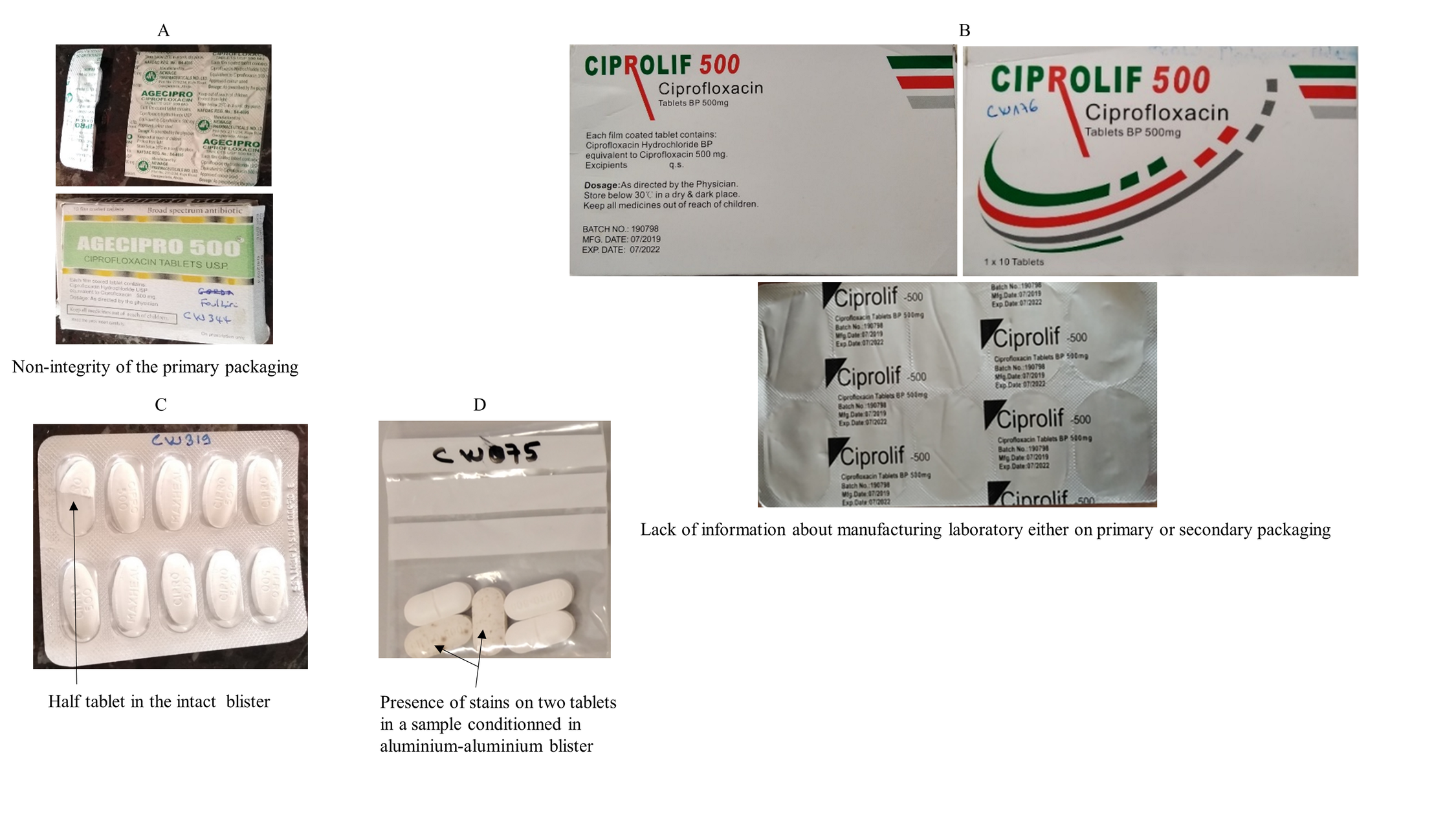

Supplement: S2 Fig — A) Sample with internal packaging cut in two parts; B) Sample with lack of information about manufacturing laboratory either on primary or secondary packaging; C) Sample with a half tablet in an intact blister; D) Sample with tablets having stains. (TIF) [file pone.0289865.s002.tif]

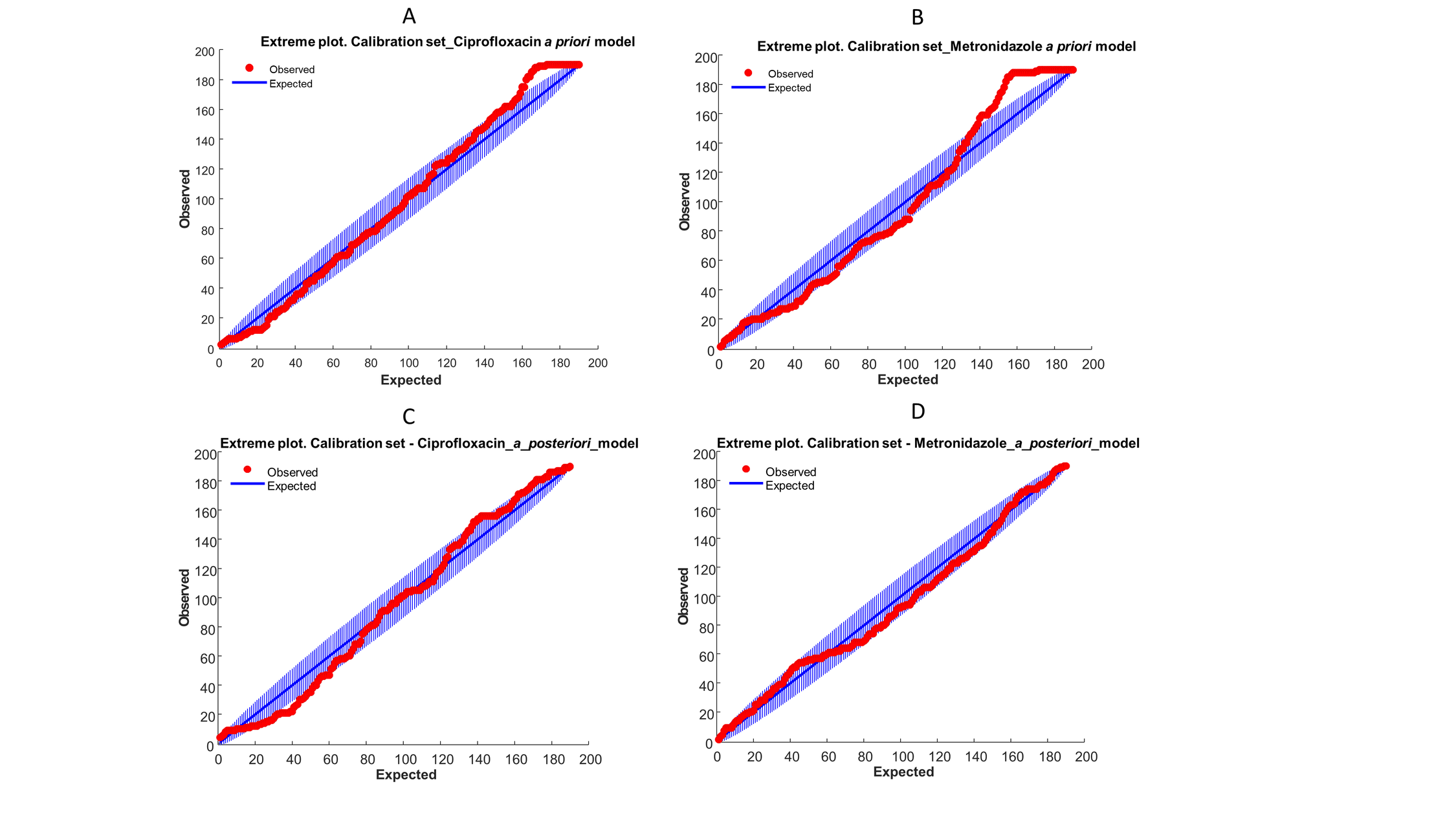

Supplement: S3 Fig — A) extreme plot of Ciprofloxacin a priori model; B) Extreme plot of Ciprofloxacin a posteriori model; C) Extreme plot of Metronidazole a priori model; D) Extreme plot of Metronidazole a posteriori model. The red dots represent samples of the calibration set and the blue vertical lines delimit the tolerance area. (TIF) [file pone.0289865.s003.tif]

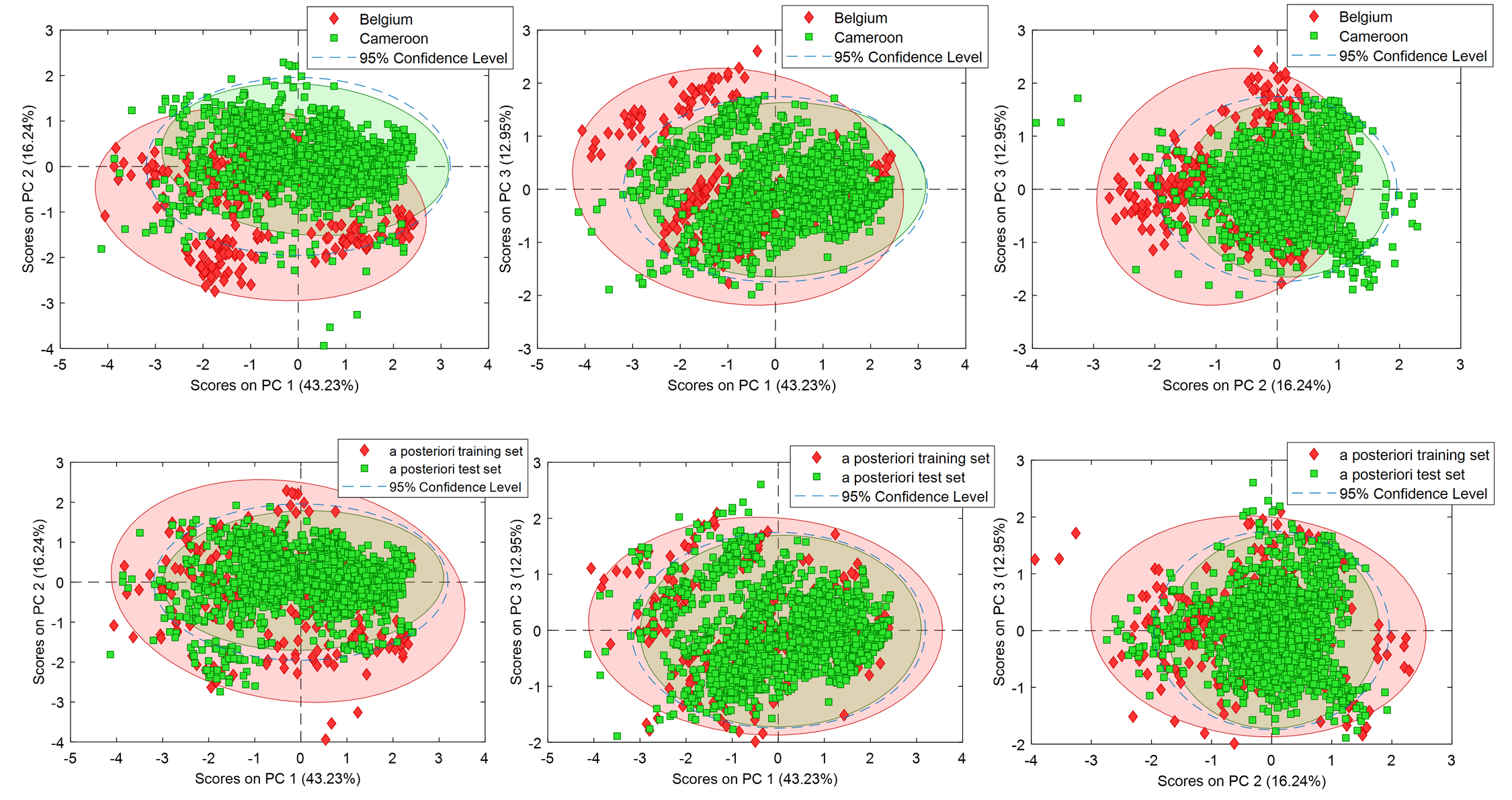

Supplement: S4 Fig — A represents PC1 vs PC2, PC1 vs PC3 and PC2 vs PC3 plots on spectra collected in Belgium (red) and Cameroon (green). B represents PC1 vs PC2, PC1 vs PC3 and PC2 vs PC3 on a posteriori training (red) and test sets (green) obtained with Kennard-Stone algorithm. (TIF) [file pone.0289865.s004.tif]

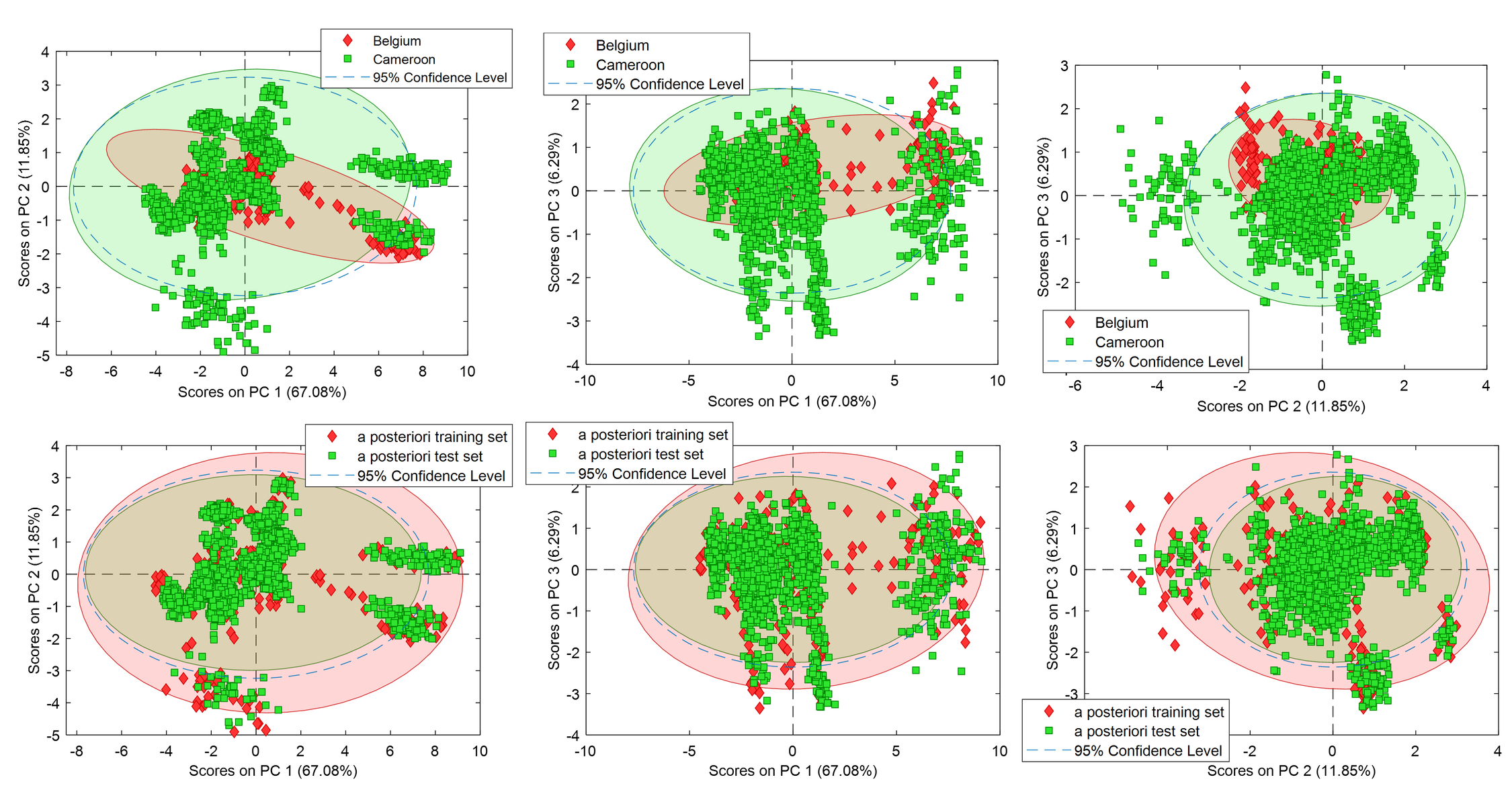

Supplement: S5 Fig — A represents PC1 vs PC2, PC1 vs PC3 and PC2 vs PC3 plots on spectra collected in Belgium (red) and Cameroon (green). B represents PC1 vs PC2, PC1 vs PC3 and PC2 vs PC3 on a posteriori training (red) and test (green) sets (green) obtained with Kennard-Stone algorithm. (TIF) [file pone.0289865.s005.tif]

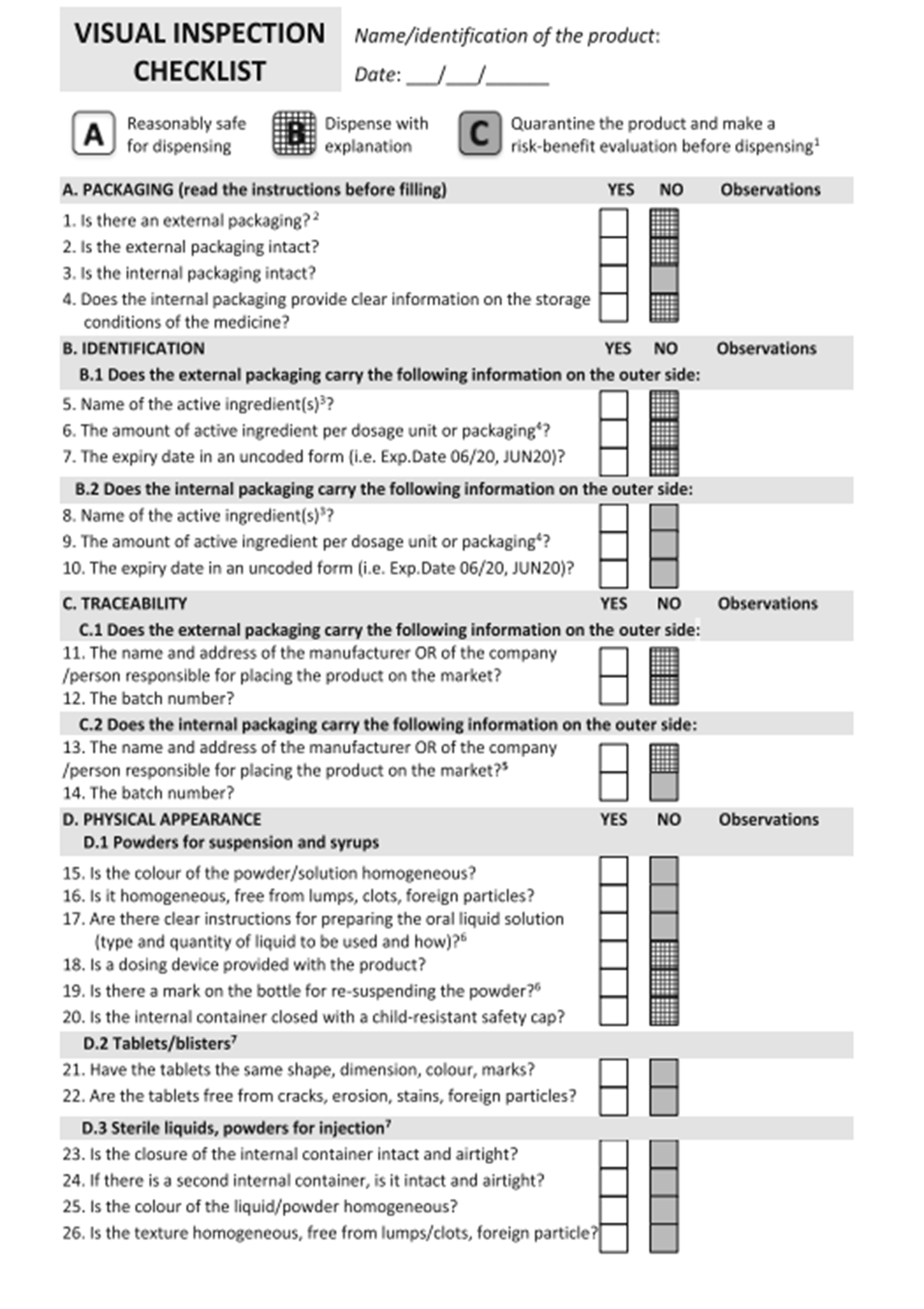

Supplement: S6 Fig — (TIF) [file pone.0289865.s006.tif]
