# Supplementary material for: Usefulness of medicine screening tools in the frame of pharmaceutical post-marketing surveillance
Source: PLoS One. 2023 Aug 11;18(8):e0289865. doi: 10.1371/journal.pone.0289865 (PMC10420354; doi:10.1371/journal.pone.0289865)
Supplement: S1 Table — (DOCX) [file pone.0289865.s007.docx]

S1 Table: List of ciprofloxacin and metronidazole samples collected for the construction of DD-SIMCA models (calibration and validation sets).

| **Stated continent of origin** | **Stated country of origin** | **Stated manufacturer** | **INN** | **Stated product name** | **Batch Number** | **N** |
| --- | --- | --- | --- | --- | --- | --- |
| **Africa** | **Senegal** | Winthrop Pharma Sanofi | Metronidazole | Flagyl 500 | 0259 | 1 |
|  |  |  | Metronidazole | Flagyl 500 | 0394 | 1 |
|  |  |  | Metronidazole | Flagyl 500 | 9887 | 1 |
|  |  |  | Metronidazole | Flagyl 500 | 0580 | 1 |
|  |  |  | Metronidazole | Flagyl 500 | 0693 | 1 |
|  |  |  | Metronidazole | Flagyl 500 | 0636 | 1 |
|  | **Cameroon** | Africure Pharmaceuticals | Metronidazole | Metronidazole BP | G096 | 1 |
|  |  |  | Metronidazole | Metronidazole BP | G097 | 1 |
|  |  |  | Metronidazole | Metronidazole BP | G137 | 1 |
|  |  |  | Metronidazole | Metronidazole BP | G272 | 1 |
|  |  |  | Metronidazole | Metronidazole BP | G149 | 1 |
|  |  |  | Ciprofloxacin | Ciprofloxacin USP | 4017005 | 1 |
|  |  |  | Ciprofloxacin | Ciprofloxacin USP | 4017004 | 2 |
|  |  |  | Ciprofloxacin | Ciprofloxacin USP | 4017003 | 1 |
|  |  |  | Ciprofloxacin | Ciprofloxacin USP | 4017002 | 1 |
|  |  |  | Ciprofloxacin | Ciprofloxacin USP | G073 | 1 |
|  | **Maroc** | Pharma 5 | Metronidazole | Nidazole | 5348 | 2 |
|  |  |  | Metronidazole | Nidazole | 5355 | 1 |
|  |  |  | Metronidazole | Nidazole | 6219 | 2 |
|  |  |  | Metronidazole | Nidazole | 7866 | 1 |
|  |  |  | Metronidazole | Nidazole | 5351 | 1 |
| **Europe** | **France** | Bailly Creat | Metronidazole | Metronidazole 500 | 150 | 2 |
|  |  |  | Metronidazole | Metronidazole 500 | 173 | 1 |
|  |  |  | Metronidazole | Creazole | 157 | 1 |
|  |  |  | Metronidazole | Metronidazole 500 | 148 | 1 |
|  |  |  | Metronidazole | Metronidazole 500 | 208 | 1 |
|  |  |  | Metronidazole | Metronidazole 500 | 160 | 1 |
|  |  |  | Metronidazole | Metronidazole 500 | 149 | 1 |
|  | **Germany** | allphamed PHARBIL Arzeimittel | Ciprofloxacin | Cipro Denk 500 | 21374 | 1 |
|  |  |  | Ciprofloxacin | Cipro Denk 500 | 21375 | 1 |
|  |  |  | Ciprofloxacin | Cipro Denk 500 | 21012 | 1 |
|  |  |  | Ciprofloxacin | Cipro Denk 500 | 20802 | 1 |
|  |  |  | Ciprofloxacin | Cipro Denk 500 | 20804 | 1 |
|  |  |  | Ciprofloxacin | Cipro Denk 500 | 21013 | 1 |
|  | **Austria** | Merck & Co | Metronidazole | Supplin 500 | B0400068 | 1 |
|  | **United Kingdom** | Betr Pharma | Ciprofloxacin | BETCIP 500 | 171229 | 1 |
| **Asia** | **India** | MAXTAR BIO-GENICS | Ciprofloxacin | Cipex 500 | MXTDR1805 | 2 |
|  |  | Medicamen Biotech | Metronidazole | Metronidazole 500 | EHT17467 | 1 |
|  |  |  | Ciprofloxacin | Ciprofloxacin USP | NT7489 | 1 |
|  |  |  | Ciprofloxacin | Ciprofloxacin USP | EHT17364 | 1 |
|  |  |  | Ciprofloxacin | Ciprofloxacin USP | EHT17367 | 1 |
|  |  |  | Ciprofloxacin | Ciprofloxacin USP | NT7491 | 1 |
|  |  | Gracure pharmaceuticals | Ciprofloxacin | Fluoxine 500 | TE-6535-1 | 1 |
|  |  |  | Ciprofloxacin | Fluoxine 500 | TE-6104 | 1 |
|  |  |  | Ciprofloxacin | Fluoxine 500 | TE-6328 | 1 |
|  |  |  | Ciprofloxacin | Fluoxine 500 | TE-6721-1 | 3 |
|  |  | Sun Pharma | Ciprofloxacin | Cifran 500 | 2909410 | 1 |
|  |  |  | Ciprofloxacin | Cifran 500 | 2917094 | 1 |
|  |  |  | Ciprofloxacin | Cifran 500 | 2905748 | 1 |
| **America** | **British West Indies** | Prost Pharma | Metronidazole | Metrole 500 | B180515 | 1 |
| **Total** | | | | | | **56** |
