# Supplementary material for: Usefulness of medicine screening tools in the frame of pharmaceutical post-marketing surveillance
Source: PLoS One. 2023 Aug 11;18(8):e0289865. doi: 10.1371/journal.pone.0289865 (PMC10420354; doi:10.1371/journal.pone.0289865)
Supplement: S2 Table — (DOCX) [file pone.0289865.s008.docx]

S2 Table: List of samples used for the specificity evaluation of DD-SIMCA models and PADs.

| **Stated continent of origin** | **Stated country of origin** | | **Stated manufacturer** | **INN** | **Family** | **Stated product name** | **Batch Number** | **N** |
| --- | --- | --- | --- | --- | --- | --- | --- | --- |
| **Asia** | **India** | | Microlabs Limited | Moxifloxacin Hydrochloride | Fluroquinolones | Micromox-400 | MFCH0002 | 1 |
|  |  |  | Cadila Pharmaceuticals | Secnidazole | Nitro-imidazoles | Amtiba | AM69E8008 | 1 |
|  |  |  | Gracure pharmaceuticals | Tinidazole | Nitro-imidazoles | Tinazol | TE-6875 | 1 |
|  | **Pakistan** | | Nabiqasim Industries | Ofloxacin | Fluroquinolones | Ketaflox | ALA001 | 1 |
| **Europe** | **France** | | Laboratoires SERB | Ornidazole | Nitro-imidazoles | Tiberal | 2511 | 1 |
|  |  |  | Sanofi | Ofloxacin | Fluroquinolones | Oflocet | 7T73E | 1 |
|  | **Spain** | | Cinfa | Norfloxacin | Fluroquinolones | Norfloxine | 1277 | 1 |
|  | | **Belgium** | Laboratory of Pharmaceutical Technology and Biopharmacy of University of Liege | Placebo (Talc, starch, silicone dioxide, Magnesium stearate) | - | - | - | 1 |
| **Total** | | | | | | | | 8 |
