# Supplementary material for: Usefulness of medicine screening tools in the frame of pharmaceutical post-marketing surveillance
Source: PLoS One. 2023 Aug 11;18(8):e0289865. doi: 10.1371/journal.pone.0289865 (PMC10420354; doi:10.1371/journal.pone.0289865)
Supplement: S3 Table — (DOCX) [file pone.0289865.s009.docx]

S3 Table: Test set samples concerned about outliers with OCC models

| N° sample | INN | Brand name | Number of spectra out (*a priori* model) | Number of spectra in (*a priori* model) | Status (*a priori* model) | Number of spectra out (*a posteriori* model) | Number of spectra in (*a posteriori* model) | Final status |
| --- | --- | --- | --- | --- | --- | --- | --- | --- |
| CW021 | Ciprofloxacin HCl | Medicamen Biotech | 0 | 10 | Conform | 1 | 9 | Conform |
| CW025 | Ciprofloxacin HCl | Cipro Denk | 0 | 10 | Conform | 2 | 8 | Conform |
| CW034 | Metronidazole | Bailly creat | 0 | 10 | Conform | 4 | 6 | Conform |
| CW046 | Metronidazole | Bailly creat | 0 | 10 | Conform | 1 | 9 | Conform |
| CW095 | Ciprofloxacin HCl | Cipronat | 0 | 10 | Conform | 1 | 9 | Conform |
| CW121 | Metronidazole | Medzol 3N (Reyoung) | 10 | 0 | Suspicious | 0 | 10 | Conform |
| CW129 | Metronidazole | Supplin 500 | 6 | 4 | Suspicious | 0 | 10 | Conform |
| CW132 | Metronidazole | Bailly creat | 1 | 9 | Conform | 0 | 10 | Conform |
| CW149 | Metronidazole | Bailly creat | 2 | 8 | Conform | 0 | 10 | Conform |
| CW153 | Metronidazole | Medzol 3N (Reyoung) | 9 | 1 | Suspicious | 0 | 10 | Conform |
| CW158 | Metronidazole | Medzol 3N (Reyoung) | 10 | 0 | Suspicious | 0 | 10 | Conform |
| CW159 | Metronidazole | Supplin 500 | 3 | 7 | Conform | 0 | 10 | Conform |
| CW161 | Metronidazole | Bailly creat | 1 | 9 | Conform | 1 | 9 | Conform |
| CW163 | Metronidazole | Bailly creat | 2 | 8 | Conform | 0 | 10 | Conform |
| CW164 | Metronidazole | Supplin 500 | 6 | 4 | Suspicious | 2 | 8 | Conform |
| CW196 | Ciprofloxacin HCl | Cipflacin | 1 | 9 | Conform | 0 | 10 | Conform |
| CW229 | Metronidazole | Medzol 3N (Huanzong) | 5 | 5 | Suspicious | 0 | 10 | Conform |
| CW230 | Metronidazole | Metronol | 8 | 2 | Suspicious | 0 | 10 | Conform |
| CW237 | Metronidazole | Metronol | 9 | 1 | Suspicious | 0 | 10 | Conform |
| CW238 | Metronidazole | Metronol | 10 | 0 | Suspicious | 0 | 10 | conform |
| CW255 | Metronidazole | Metro 500 | 1 | 9 | Conform | 0 | 10 | Conform |
| CW257 | Metronidazole | Metronol | 8 | 2 | Suspicious | 0 | 10 | Conform |
| CW258 | Metronidazole | Metronol | 10 | 0 | Suspicious | 0 | 10 | Conform |
| CW260 | Metronidazole | Metronol | 8 | 2 | Suspicious | 0 | 10 | Conform |
| CW284 | Metronidazole | Medzol 3N (Huanzong) | 1 | 9 | Conform | 0 | 10 | Conform |
| CW303 | Metronidazole | Medzol 3N (Huanzong) | 1 | 9 | Conform | 0 | 10 | Conform |
| CW305 | Metronidazole | Bailly creat | 1 | 9 | Conform | 0 | 10 | Conform |
| CW306 | Metronidazole | Metronol | 10 | 0 | Suspicious | 0 | 10 | Conform |
| CW308 | Metronidazole | Bailly creat | 1 | 9 | Conform | 1 | 9 | Conform |
| CW334 | Metronidazole | Anhui BBCA | 10 | 0 | Suspicious | 0 | 10 | Conform |
| CW363 | Metronidazole | Anhui BBCA | 9 | 1 | Suspicious | 0 | 10 | Conform |
| Total | | - | 143 | 2777 | - | 13 | 2907 | - |
