# Supplementary material for: Usefulness of medicine screening tools in the frame of pharmaceutical post-marketing surveillance
Source: PLoS One. 2023 Aug 11;18(8):e0289865. doi: 10.1371/journal.pone.0289865 (PMC10420354; doi:10.1371/journal.pone.0289865)
Supplement: S4 Table — (DOCX) [file pone.0289865.s010.docx]

S4 Table: Confusion matrix of Ciprofloxacin PADs results

|  |  | **Actual class** | | **Performances** | |
| --- | --- | --- | --- | --- | --- |
|  | **Total population** | **Pass** | **Fail** | **Sensitivity** | **Specificity** |
| **Predicted class** | **Pass** | 150 (TP) | 3 (FP) | 100.0% | 40.0% |
|  | **Fail** | 0 (FN) | 2 (TN) |  |  |

Legend: TP=true positives; FP= false positives; FN=false negatives; TN=true negatives
