# Supplementary material for: Usefulness of medicine screening tools in the frame of pharmaceutical post-marketing surveillance
Source: PLoS One. 2023 Aug 11;18(8):e0289865. doi: 10.1371/journal.pone.0289865 (PMC10420354; doi:10.1371/journal.pone.0289865)
Supplement: S1 File — (DOCX) [file pone.0289865.s011.docx]

**S1 File: NIR supplementary data**

**NIR-S-G-1 instrument settings:**

Scan config: “column 1”; Digital resolution: 228; Max resolution: 353; Pattern :228; Total pattern: 228; Start wavelength: 900nm; End wavelength: 1700nm; Pattern width: 7.03nm; Exposure time: 0.635ms; PGA gain: 64; Average number of scans: 6.

Supplementary information about spectra acquisition are available in the raw data of spectra files on the repository: <https://osf.io/ht2k9/files/osfstorage>

**Bootstrapping strategy:**

To compute the confidence intervals, we used a bootstrap strategy as followed:

1. The calibration and validation datasets were assembled in a single training dataset while keeping the test set and non-members dataset aside.
2. From the training dataset, *k=280* spectra are sampled uniformly at random, with replacement (function datasample in Matlab) to build *n=10,000* bootstrap training datasets.
3. Each bootstrap training dataset is then split into calibration and validation set with the Kennard and Stone algorithm (190 spectra in calibration and 90 spectra in validation datasets).
4. *n* DD-SIMCA models are built with the bootstrap calibration dataset using the parameters (number of principal components and α value) indicated in table 3. The bootstrap validation datasets are then projected onto these DD-SIMCA models. Using these data, the median sensitivities for calibration and validation are computed and their 95% confidence interval are estimated by the by the 2.5th and 97.5th percentile of the empirical distribution of sensitivities. These values are reported in table 3.
5. The test set and the non-member datasets are projected onto each bootstrap DD-SIMCA models. Their sensitivities and number of rejected models are computed applying the acceptance rule for samples (maximum 4 out spectra authorized). Their median sensitivity and the 95% confidence interval are estimated by the by the 2.5th and 97.5th percentile of the empirical distribution of sensitivities are reported in table 3.
6. The whole strategy is repeated for the a priori and the a posteriori data split of the two studied molecules.

**Definition of concepts:**

A priori model: model built without any further knowledge of the representativeness of the training set in relation to the test set (samples from the field study).

A posteriori model: models issued from the hypothesis of the representativity of the training set in relation with de test set.
