# Supplementary material for: Usefulness of medicine screening tools in the frame of pharmaceutical post-marketing surveillance
Source: PLoS One. 2023 Aug 11;18(8):e0289865. doi: 10.1371/journal.pone.0289865 (PMC10420354; doi:10.1371/journal.pone.0289865)
Supplement: S2 File — (DOCX) [file pone.0289865.s012.docx]

**S2 File: PADreader supplementary information**

PADreader is normally used to collect sample metadata and to obtain a properly focused, oriented, and scaled image of the physical PAD card. The app then applies chemometric algorithms to classify the results of the PAD test. The raw data and test results are stored locally on the phone and uploaded to a central database when there is a WiFi connection. In this study, we attempted to use PADreader to “read” PAD images displayed on a computer monitor. This was potentially a difficult task because some of the images had varying orientations, keystone or rotational distortions, poor focus, or ink marks near or on the fiducial marks, and because the brightness and color appearance of the images displayed on a computer screen differs from the brightness and color appearance of the physical cards. Several attempts were made to apply other data analytics models for concentration determination in the successfully captured images, but they were unsuccessful, consistent with the failure of efforts to read the concentration “by eye”. The likely reason for both of these failures is that when the user applies the ciprofloxacin powder to the PAD card, there is no control over the amount of powder applied in each lane.

The blank was read as hydroxychloroquine (with a certainty of -2.25), metronidazole as albendazole (with a certainty of -1.98) by the PADreader, which means that the neural net classifier was just guessing.
